# Supplementary material for: Discovery of a three-proton insertion mechanism in α-molybdenum trioxide leading to enhanced charge storage capacity
Source: Nat Commun. 2023 Sep 7;14:5490. doi: 10.1038/s41467-023-41277-8 (PMC10485074; doi:10.1038/s41467-023-41277-8)
Supplement: Supplementary file 1 — Supplementary Information [file 41467_2023_41277_MOESM1_ESM.pdf]

Supporting Information

Discovery of a Three-proton Insertion Mechanism in α-Molybdenum Trioxide Leading to Enhanced Charge Storage Capacity

Yongjiu Lei[1#], Wenli Zhao[1#], Jun Yin[1,2#], Yinchang Ma[1], Zhiming Zhao[1], Jian Yin[1], Yusuf Khan[1], Mohamed Nejib Hedhili[3], Long Chen[3], Qingxiao Wang[3], Youyou Yuan[3], Xixiang Zhang[1], Osman M. Bakr[1], Omar F. Mohammed[4], Husam N. Alshareef [1*]

[1] Physical Science and Engineering Division, King Abdullah University of Science and Technology (KAUST), Thuwal 23955-6900, Kingdom of Saudi Arabia

[2] Department of Applied Physics, The Hong Kong Polytechnic University, Kowloon 999077 Hong Kong, P. R. China

[3] Core Labs, King Abdullah University of Science and Technology (KAUST), Thuwal 23955-6900, Kingdom of Saudi Arabia

[4] Advanced Membranes and Porous Materials Center, KAUST Catalysis Center, Physical Science and Engineering Division, King Abdullah University of Science and Technology, Thuwal 23955-6900, Kingdom of Saudi Arabia

[#] These authors contributed equally to this work

* Corresponding author: Prof. Husam Niman Alshareef (email: husam.alshareef@kaust.edu.sa)

Supplementary Figures and notes

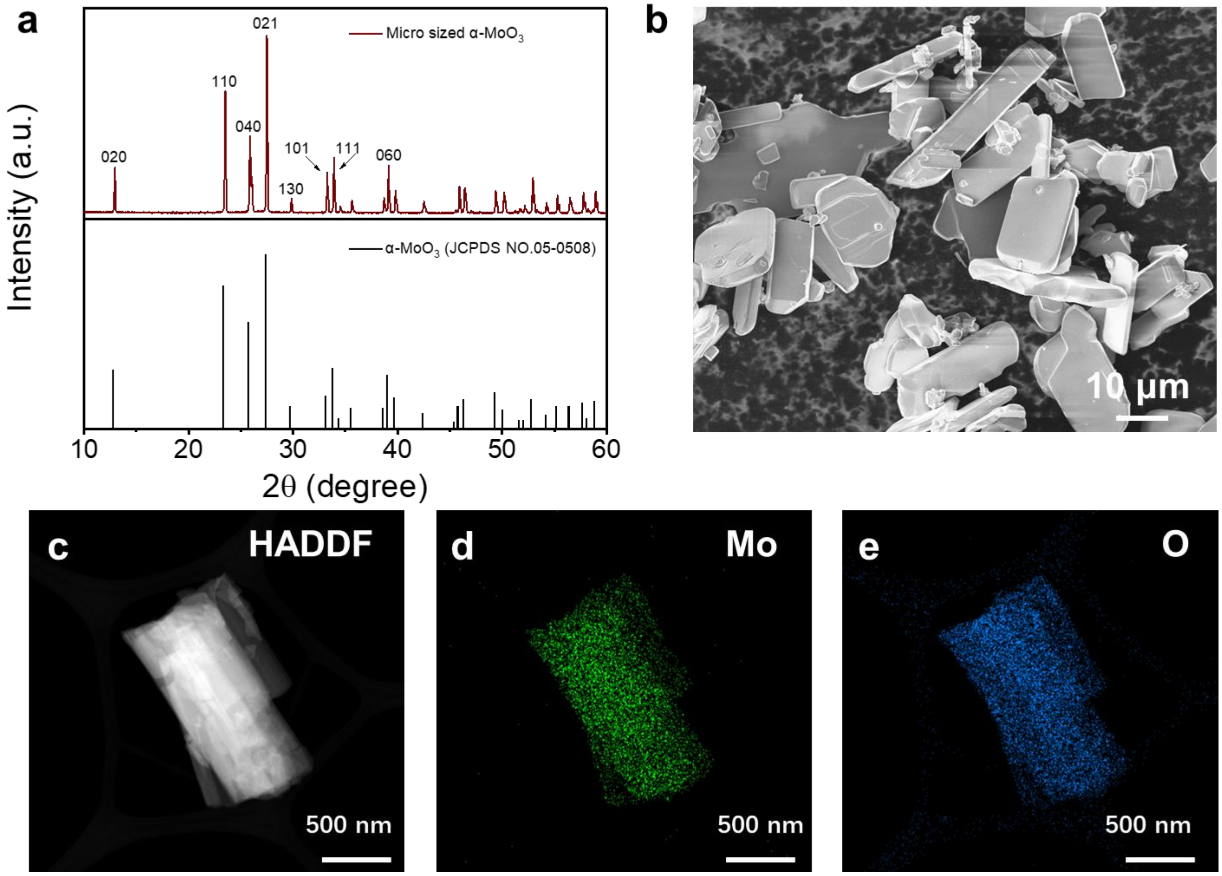

Supplementary Figure 1| Physical characterizations of the as-received α-MoO_3. **a** The X-ray diffraction (XRD) pattern and **b** the scanning electron microscopy (SEM) image of MoO_3. **c** High-angle annular dark-field image (HADDF) of MoO_3. Energy dispersive spectroscopy (EDS) for elemental mapping of **d** molybdenum and **e** oxygen.

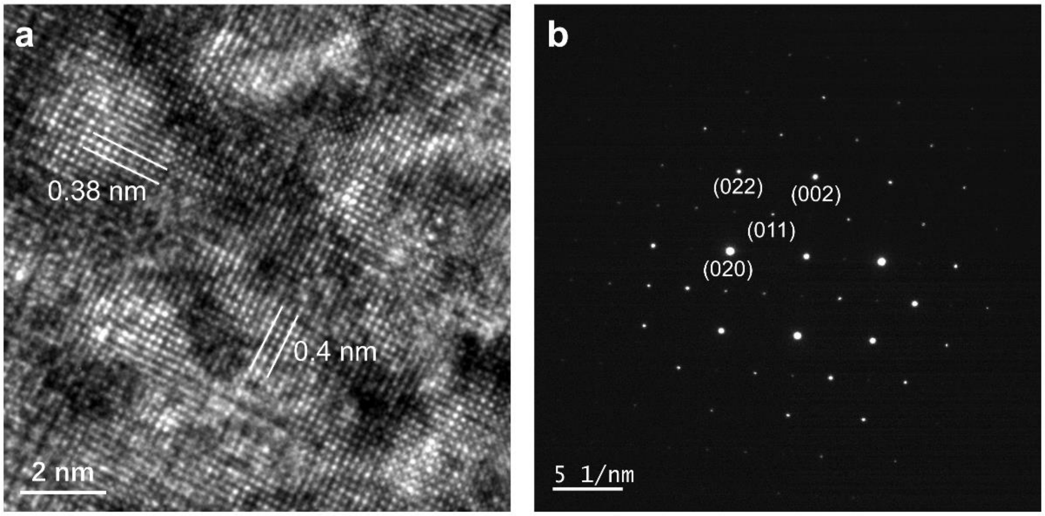

Supplementary Figure 2| Structure characterizations of the pristine α-MoO_3. a High-resolution transmission electron microscopic (HR-TEM) image MoO_3. The lattice fringe widths were estimated to be 0.4 and 0.38 nm, corresponding to the (010) and (001) plane spacings of the α-MoO_3, respectively. **b** Selected area electron diffraction (SAED) pattern with a zone axis along [100].

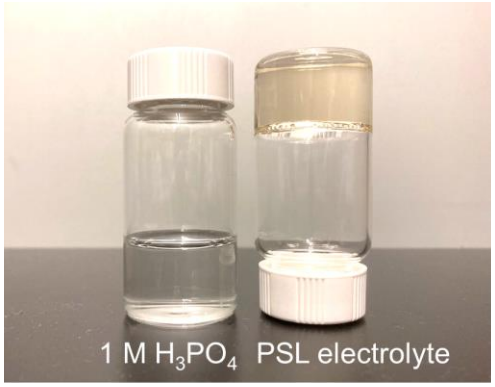

Supplementary Figure 3| Photographic photo of the H_3PO_4-based electrolytes.

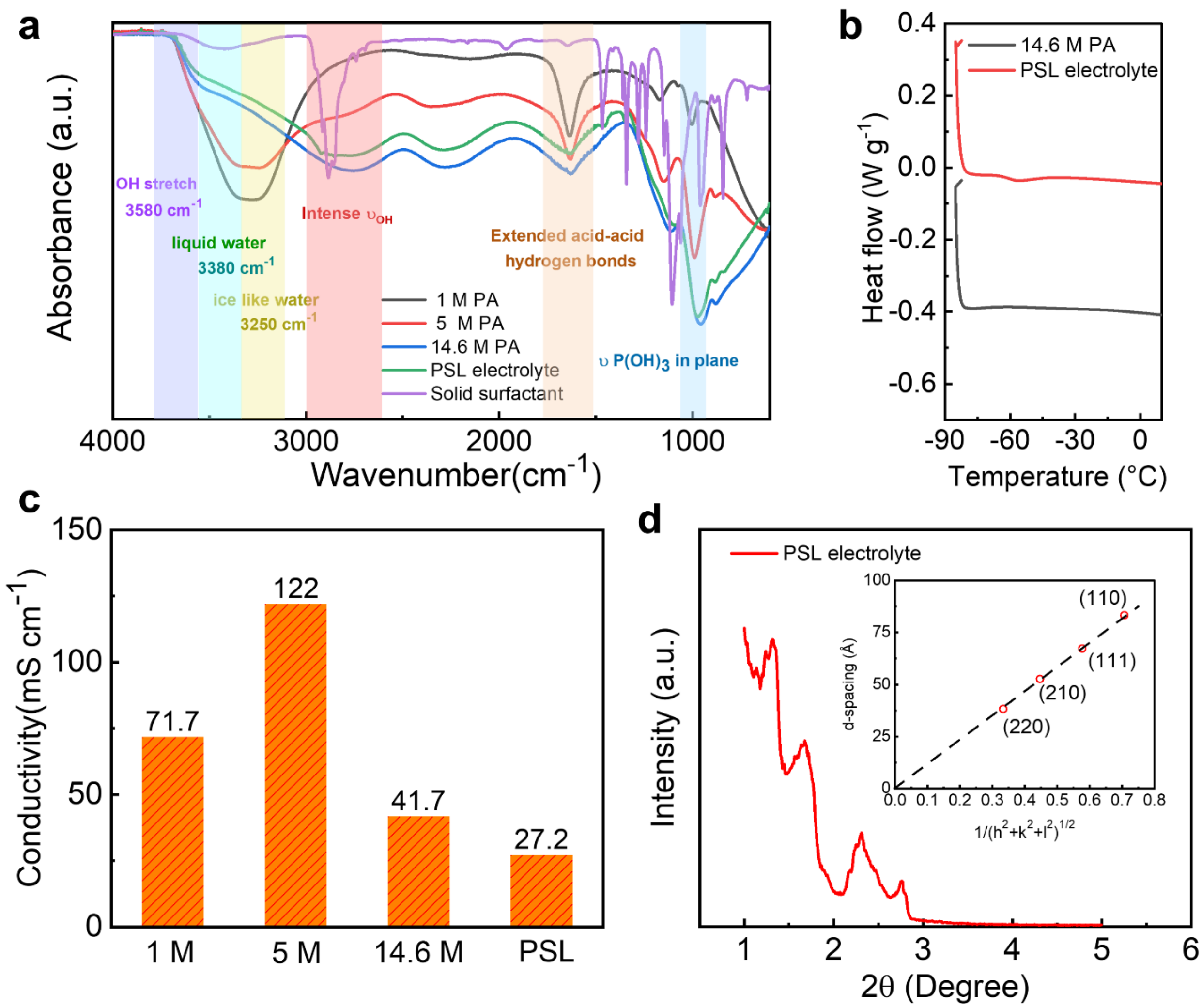

Supplementary Figure 4| Physical characterizations of the phosphoric acid surfactant lyotropic liquid crystalline (PSL) electrolyte. **a** Fourier transform infrared spectroscopy (FT-IR) of different phosphoric acid (PA) electrolytes. **b** Differential scanning calorimetry (DSC) data of different electrolytes. **c** The ionic conductivity of different electrolytes. **d** XRD pattern of the PSL electrolyte.

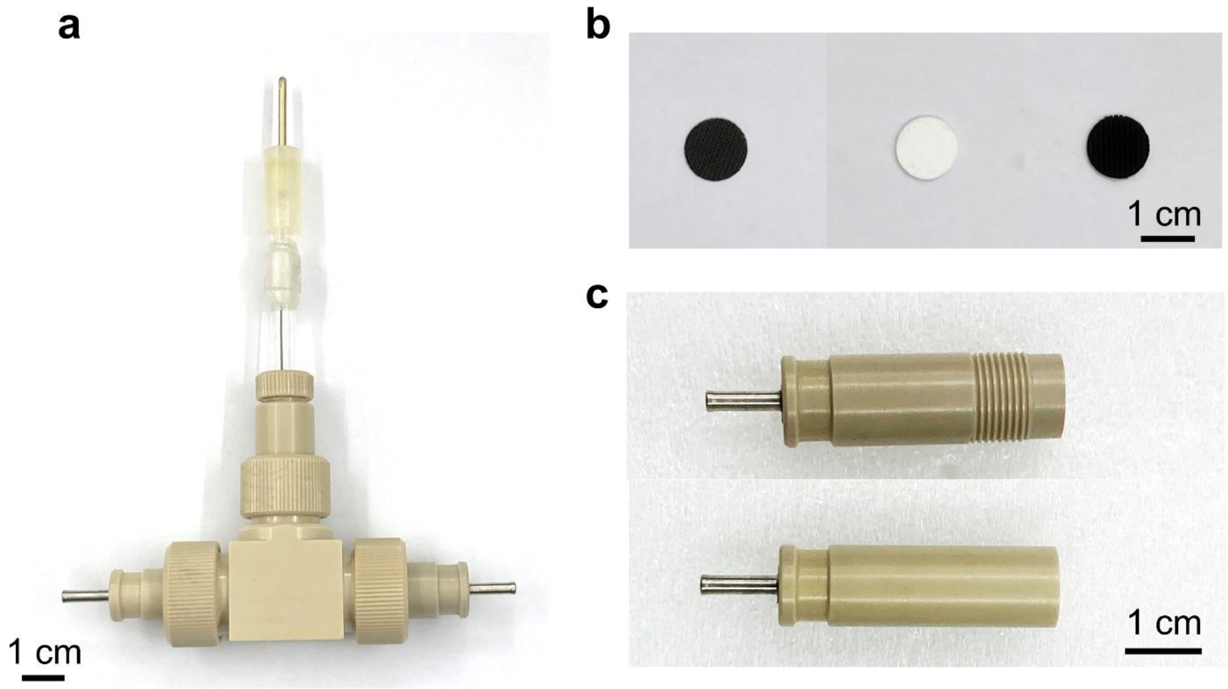

Supplementary Figure 5| Photographic photos of the Swagelok-type cell used for electrochemical testing. a The full cell. **b** The working electrode (left), separator (middle), and counter electrode (right). **c** The glass carbon electrodes that work as the current collector.

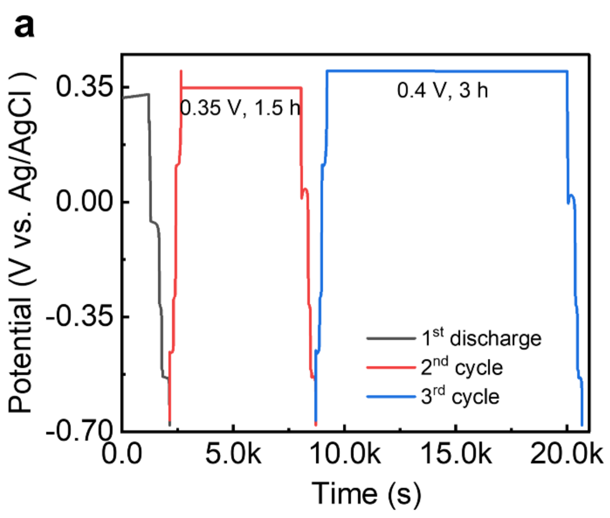

Supplementary Figure 6| The protons extraction experiment of MoO_3 electrode. a Galvanostatic charge-discharge (GCD) and potentiostatic curves (constant voltage steps during each cycle) of MoO_3 electrodes. **b** the corresponding GCD curves from **a**.

Supplementary Note: A constant voltage of 0.35 V was applied for 1.5 h after the first charge process to extract the trapped protons. After the second discharge process, a constant voltage of 0.4 V was applied for 3 h to extract the trapped protons. The MoO_3 electrode shows a similar specific discharge capacity (around 360 mA h g^{-1}) compared with the one without potentiostatic treatment. The purple oval circle in Supplementary Figure 6 **b** indicated the curves changes comparing the GCD curves without constant voltage steps treatment in Figure 2 **a**.

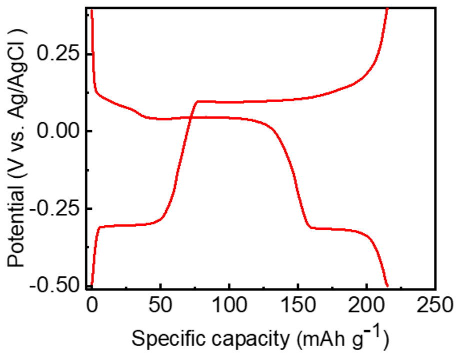

Supplementary Figure 7| The GCD curve of the MoO_3 electrode at 2 A g^{-1} via two-proton mechanism using PSL electrolyte.

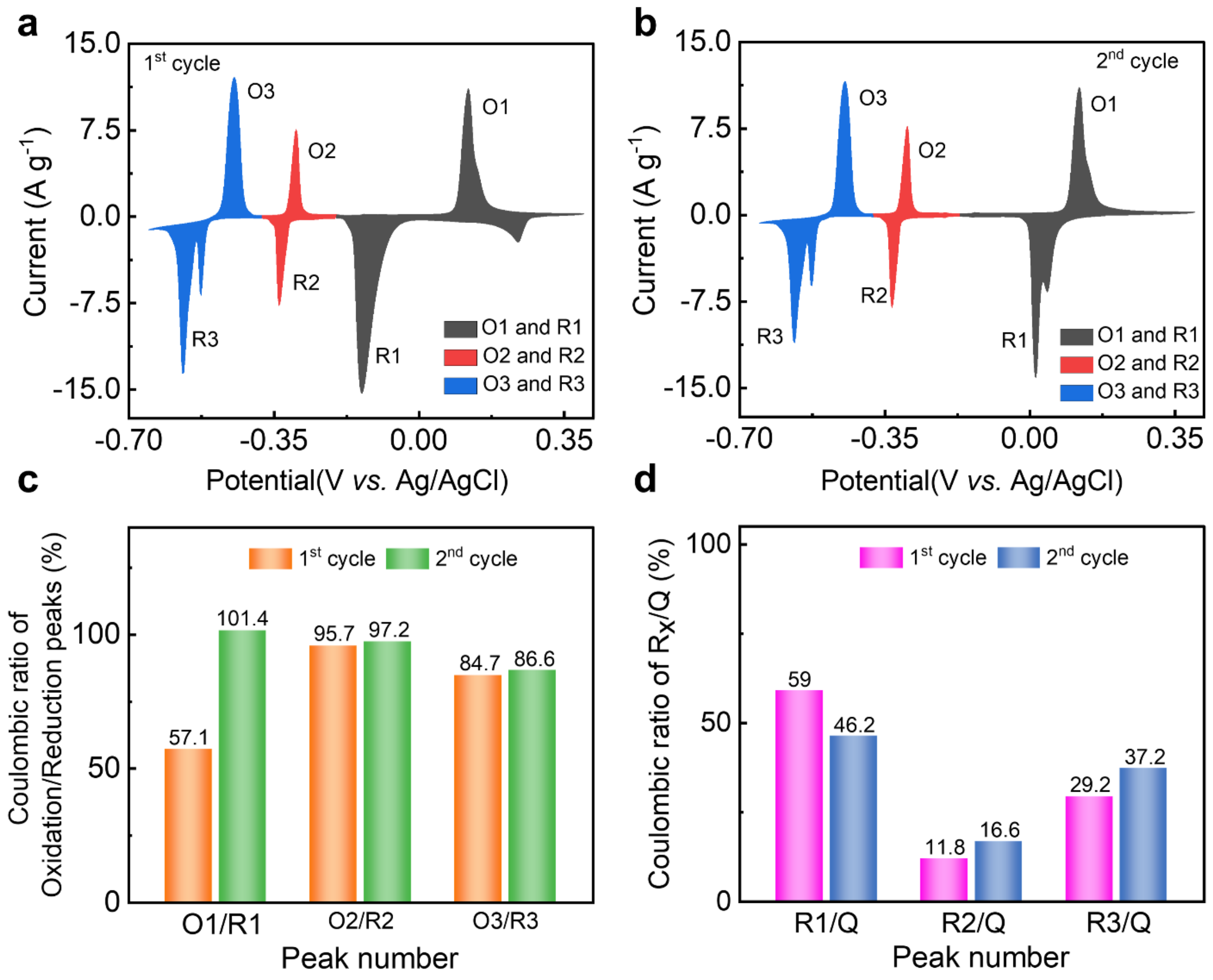

Supplementary Figure 8| Electrochemical performance of MoO_3 electrode using the PSL electrolyte. The first **a** and **b** second cyclic voltammetry (CV) curves (at 1 mV s^{-1}) of MoO_3 electrode using PSL electrolyte. The Coulombic ratio of **c** Oxidation/Reduction peaks and **d** Reduction peaks/total coulomb in the first and second CV curves of the MoO_3 electrode using PSL electrolyte.

Supplementary Note: The Coulombic ratio in Supplementary Figures 8 **c** and **d** was calculated based on the integral area of different redox pairs in Supplementary Figures 8 **a** and **b**.

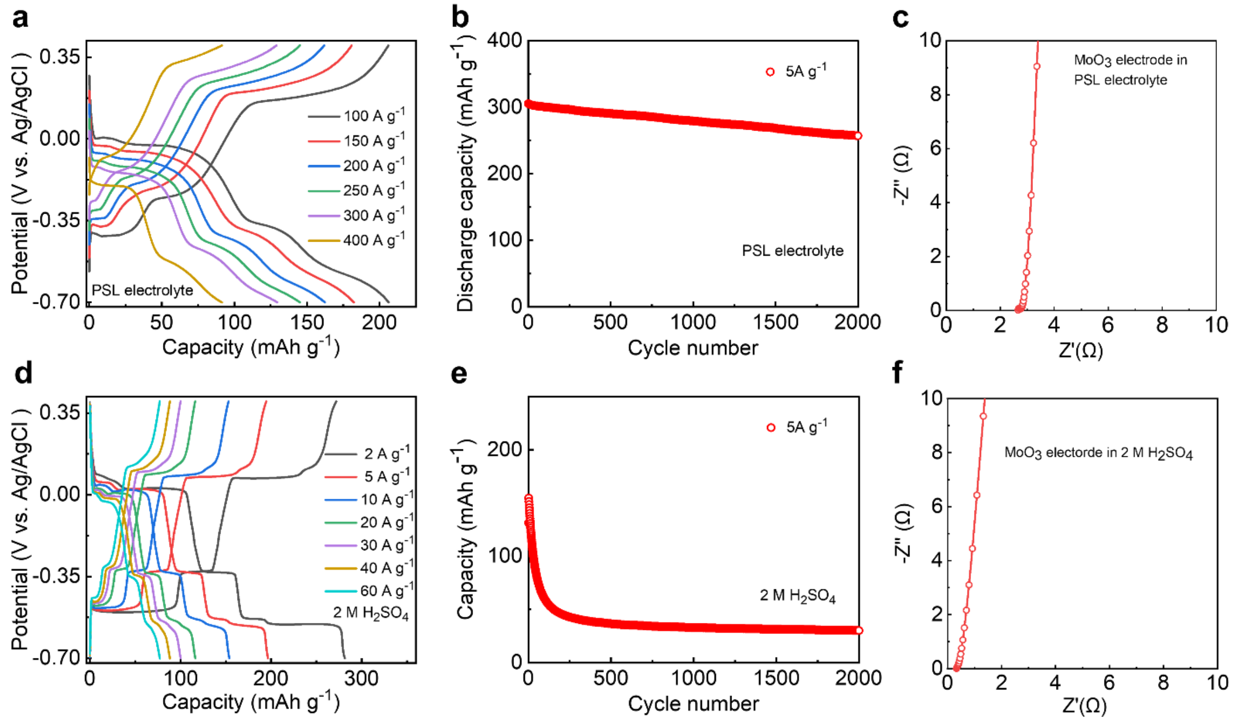

Supplementary Figure 9| Electrochemical performance of MoO_3 electrode using the different acid electrolytes. GCD curves of MoO_3 electrodes using **a** PSL electrolyte and **d** 2M H_2SO_4. Cycling performance of MoO_3 electrodes using **b** PSL electrolyte and **e** 2M H_2SO_4. Nyquist impedance plot for the MoO_3 electrodes using **c** PSL electrolyte and **f** 2M H_2SO_4.

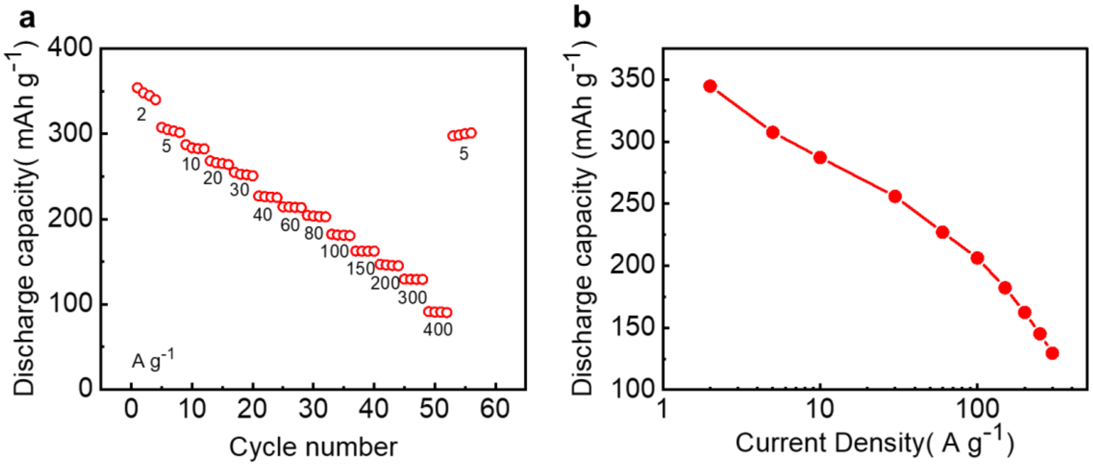

Supplementary Figure 10| Electrochemical performance of α-MoO_3 electrode using PSL electrolyte. a Rate performance of α-MoO_3 electrode at the current density of 2 to 400 A g^{-1}. **b** Specific discharge capacity of α-MoO_3 electrode at the current density of 2 to 300 A g^{-1}.

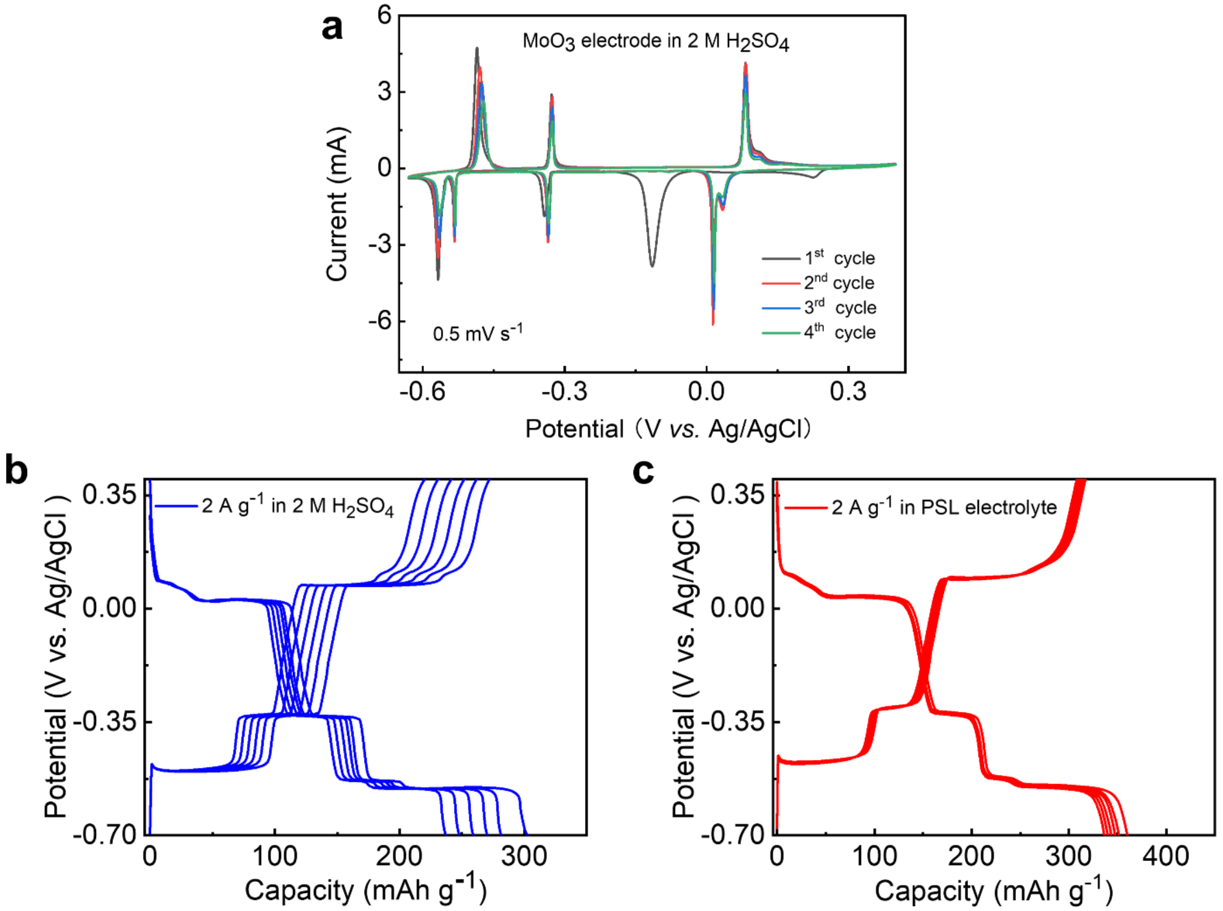

Supplementary Figure 11| Electrochemical performance of α-MoO_3 electrode using the different acid electrolytes. a The initial four cycles of CV curves of MoO_3 electrode using 2M H_2SO_4. Six cycles of GCD curves of α-MoO_3 electrodes using **b** 2M H_2SO_4 and **c** PSL electrolyte.

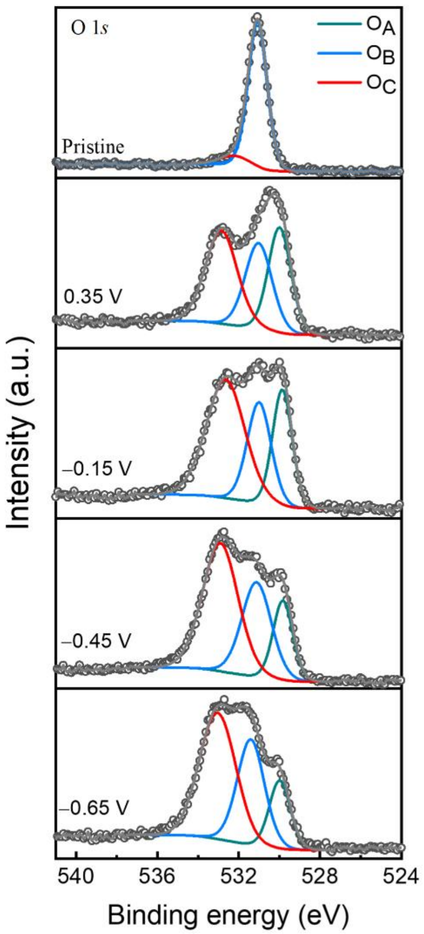

Supplementary Figure 12| The O 1*s* XPS spectrums of α-MoO_3 electrodes performed at different discharge states.

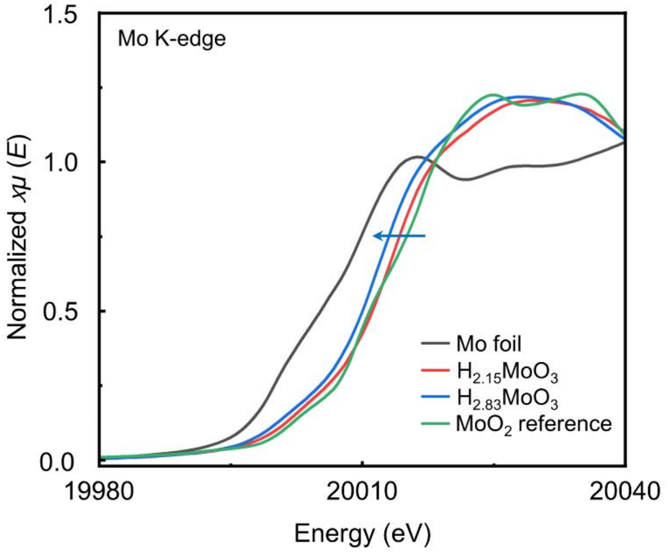

Supplementary Figure 13| The XANES spectra of $H_{2.15}MoO_3$ and $H_{2.83}MoO_3$ compared to Mo foil and MoO_2 reference.

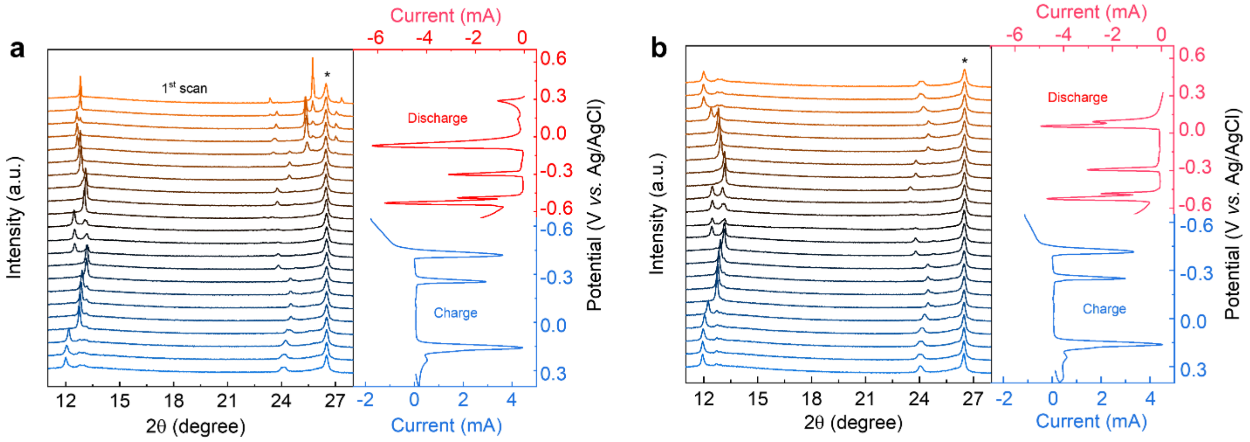

Supplementary Figure 14| In operando XRD measurements of α-MoO_3 electrodes. a The first galvanostatic discharge and charge cycle and **b** The second cycle using PSL electrolyte with a three-electrode system (Ag/AgCl worked as the reference electrode). The asterisks represent the background peaks of carbon paper.

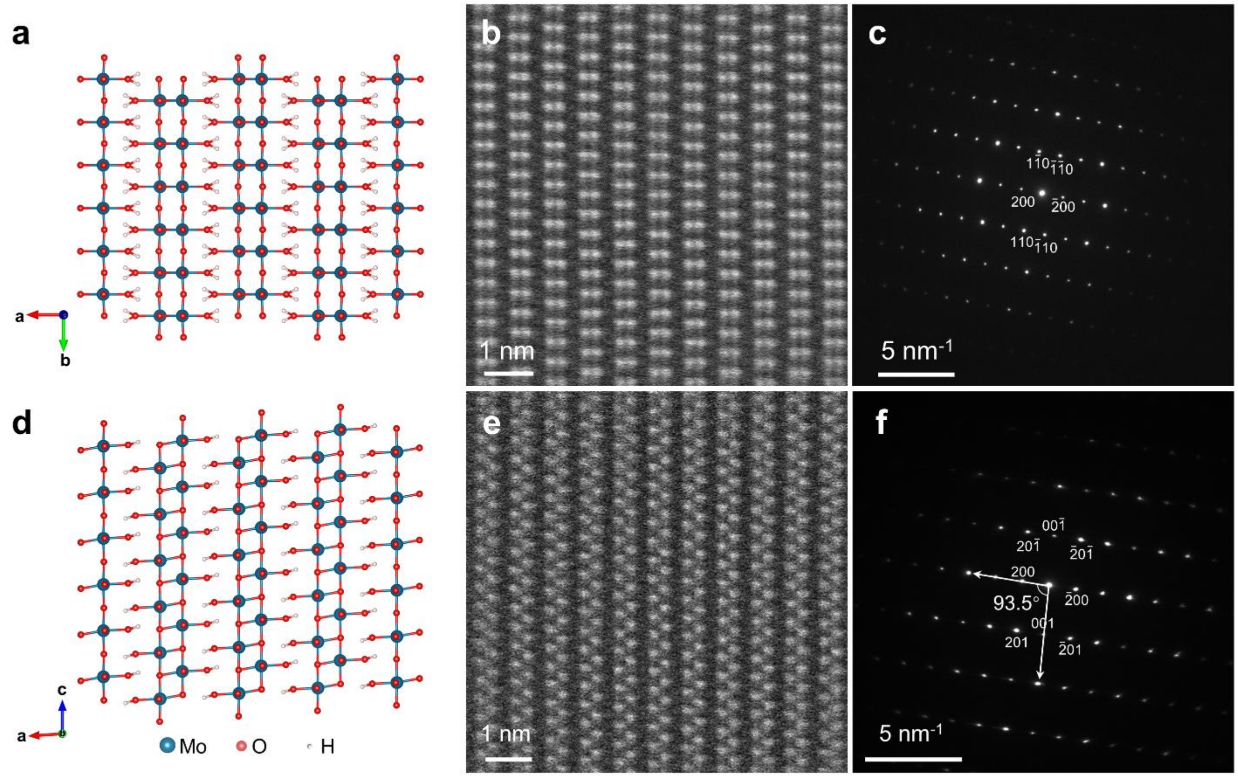

Supplementary Figure 15| Characterizations of the $H_{1.86}MoO_3$ structure. a to **c** The DFT simulated crystal structure, HR-STEM, and SAED pattern of $H_{1.86}MoO_3$ viewed along the [001] direction. **d** to **f** The DFT simulated crystal structure, HR-STEM, and SAED pattern of $H_{1.86}MoO_3$ viewed along the [010] direction.

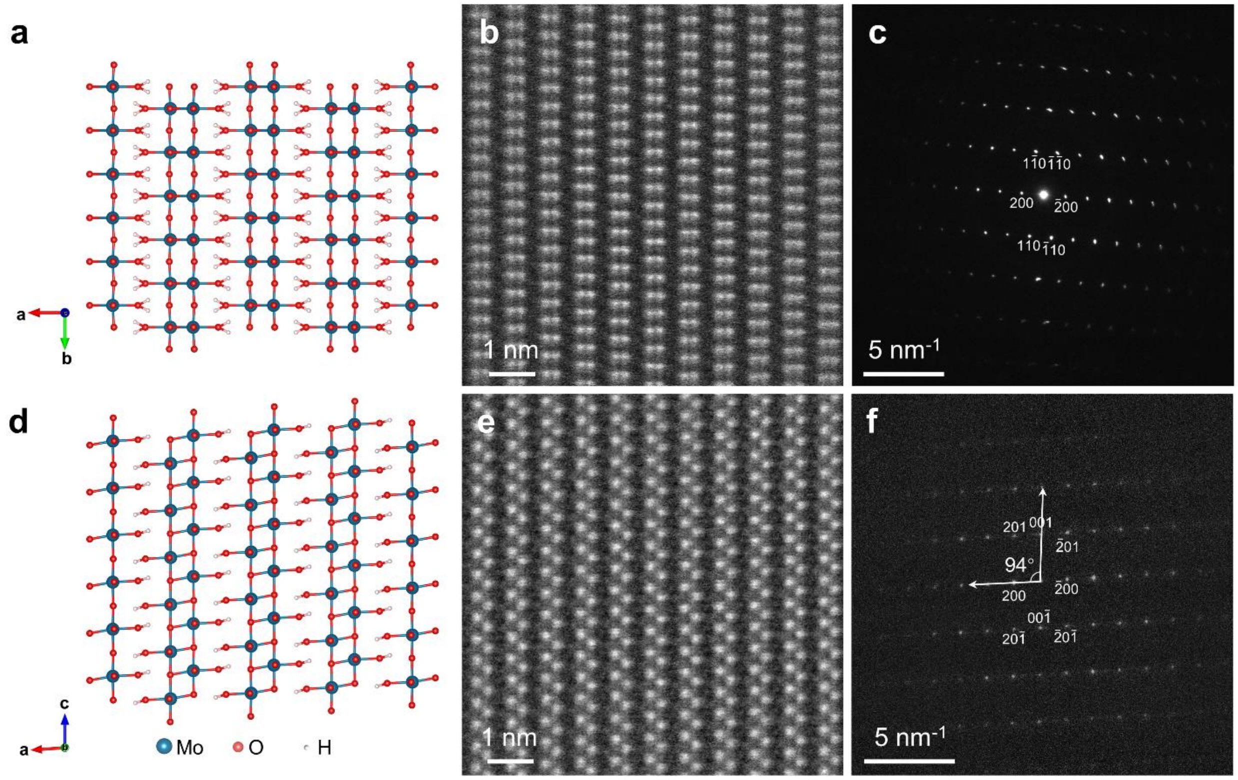

Supplementary Figure 16| Characterizations of the $H_{2.15}MoO_3$ structure. a to **c** The DFT simulated crystal structure, HR-STEM, and SAED pattern of $H_{2.15}MoO_3$ viewed along the [001] direction. **d** to **f** The DFT simulated crystal structure, HR-STEM, and SAED pattern of $H_{2.15}MoO_3$ viewed along the [010] direction.

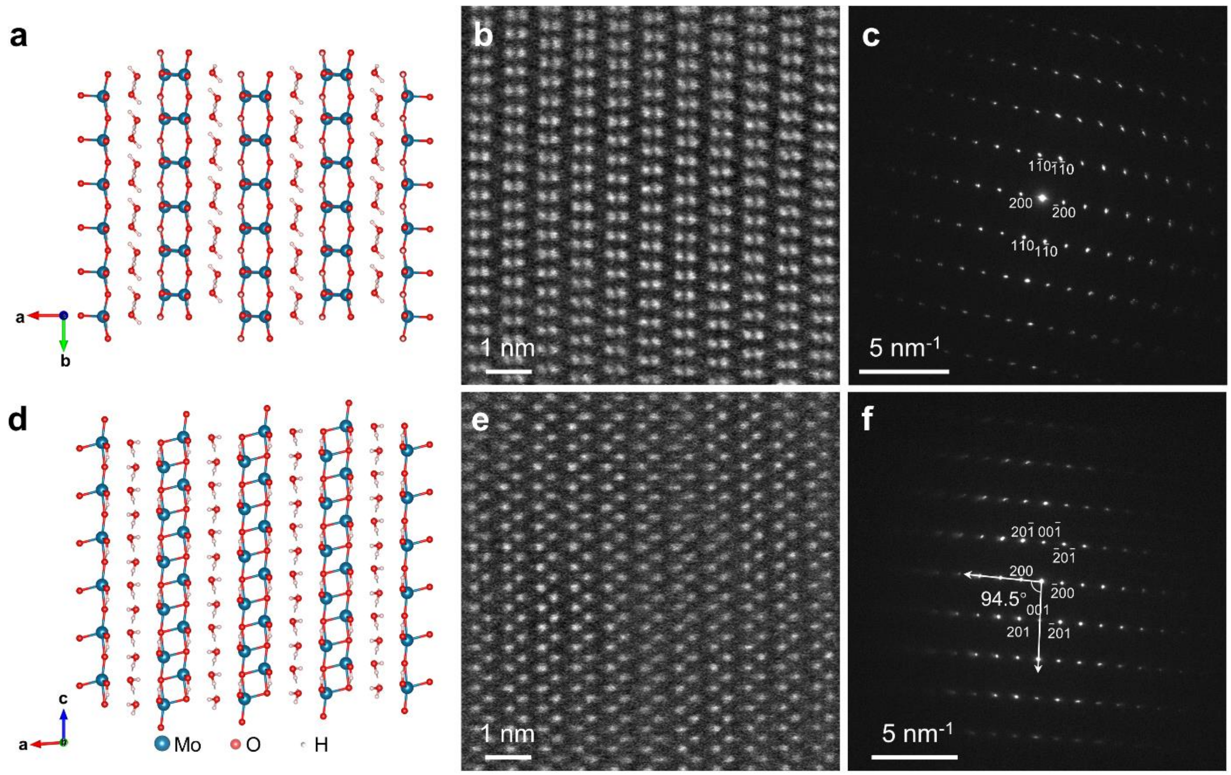

Supplementary Figure 17| Characterizations of the $H_{2.83}MoO_3$ structure. a to **c** The DFT simulated crystal structure, HR-STEM, and SAED pattern of $H_{2.83}MoO_3$ viewed along the [001] direction. **d** to **f** The DFT simulated crystal structure, HR-STEM, and SAED pattern of $H_{2.83}MoO_3$ viewed along the [010] direction.

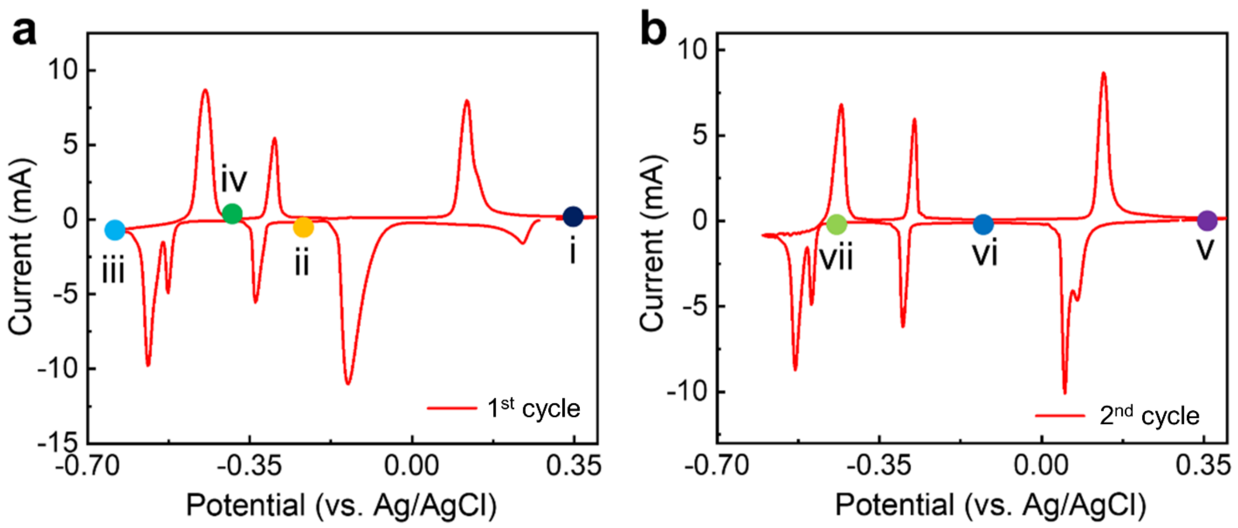

Supplementary Figure 18| CV curves of α-MoO_3 electrodes tested in a three-electrode system using PSL electrolyte. a The first cycle and **b** the second cycle. The marked point indicated the cut-off potential for ex-situ XRD tests.

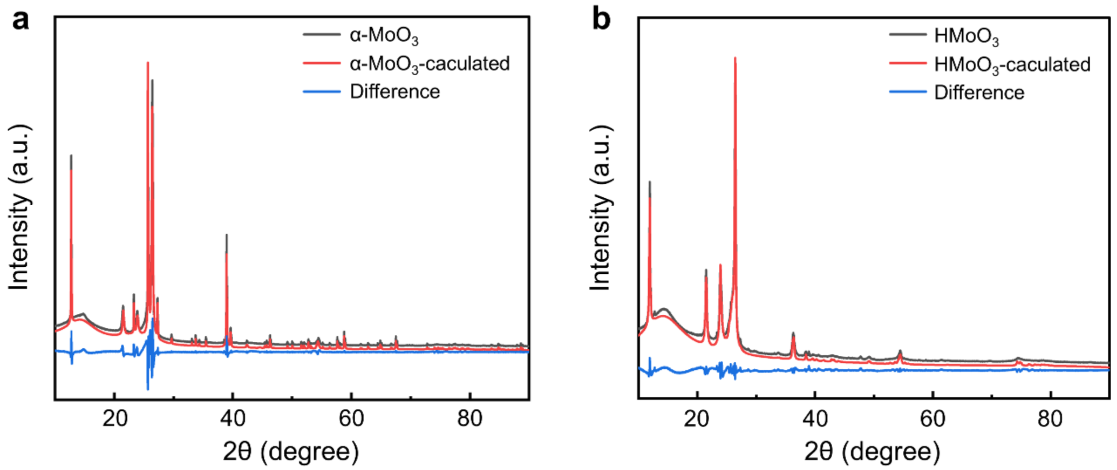

Supplementary Figure 19| The ex-situ XRD curves of α-MoO_3 before and after the first cycle. **a** The pristine α-MoO_3 and **b** $HMoO_3$.

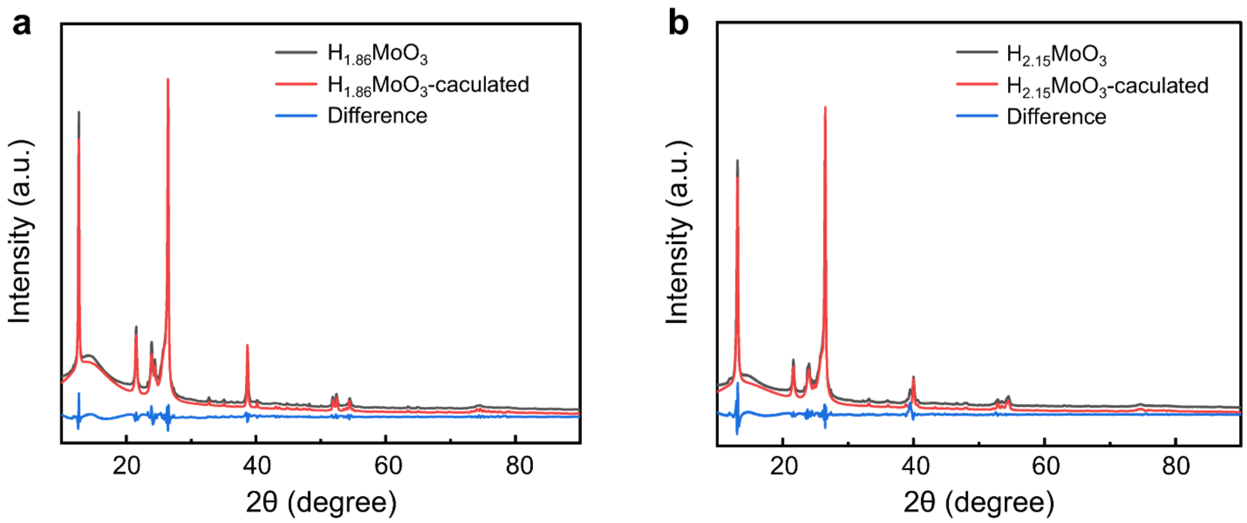

Supplementary Figure 20| The ex-situ XRD curves of α-MoO_3 during the protons (de)intercalation process. a The $H_{1.86}MoO_3$ and **b** $H_{2.15}MoO_3$.

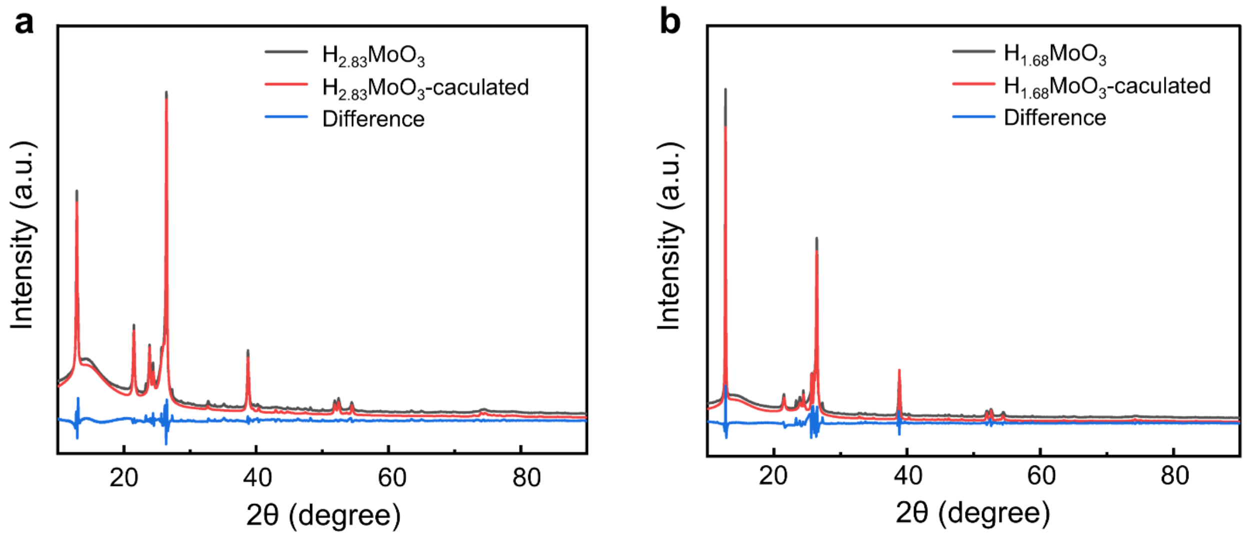

Supplementary Figure 21| The ex-situ XRD curves of α-MoO_3 during the protons (de)intercalation process. a The $H_{2.83}MoO_3$ and **b** $H_{1.68}MoO_3$.

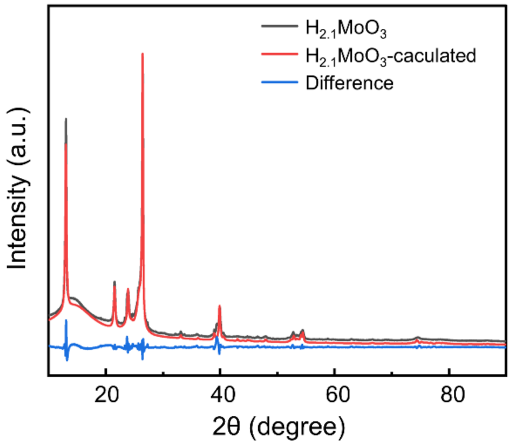

Supplementary Figure 22| The ex-situ XRD curves of α-MoO_3 during the protons (de)intercalation process($H_{2.1}MoO_3$).

Supplementary Table 1 The structural information and refined lattice parameters of H_xMoO_3 obtained from ex-situ XRD pattern refinement.

Stage	Phase	Space group	a/Å	b/Å	c/Å	Vol/Å^3	Beta/°
i	α-MoO_3	Pnma	13.8638	3.7004	3.9649	203.41	90
ii	$H_{1.68}MoO_3$	C2/m	13.9312	3.7752	4.0717	213.756	94.48
iii	$H_{2.83}MoO_3$	C	13.7179	3.8614	4.0661	214.5206	95.14
iv	$H_{2.1}MoO_3$	C	13.6057	3.8674	4.058	212.6369	95.36
v	$HMoO_3$	I2/m	14.0808	3.7163	7.4735	390.1492	93.96
vi	$H_{1.86}MoO_3$	C2/m	13.9811	3.7730	4.0516	213.2424	93.85
vii	$H_{2.15}$MoO3	C	13.5835	3.8979	4.0382	213.0686	94.78

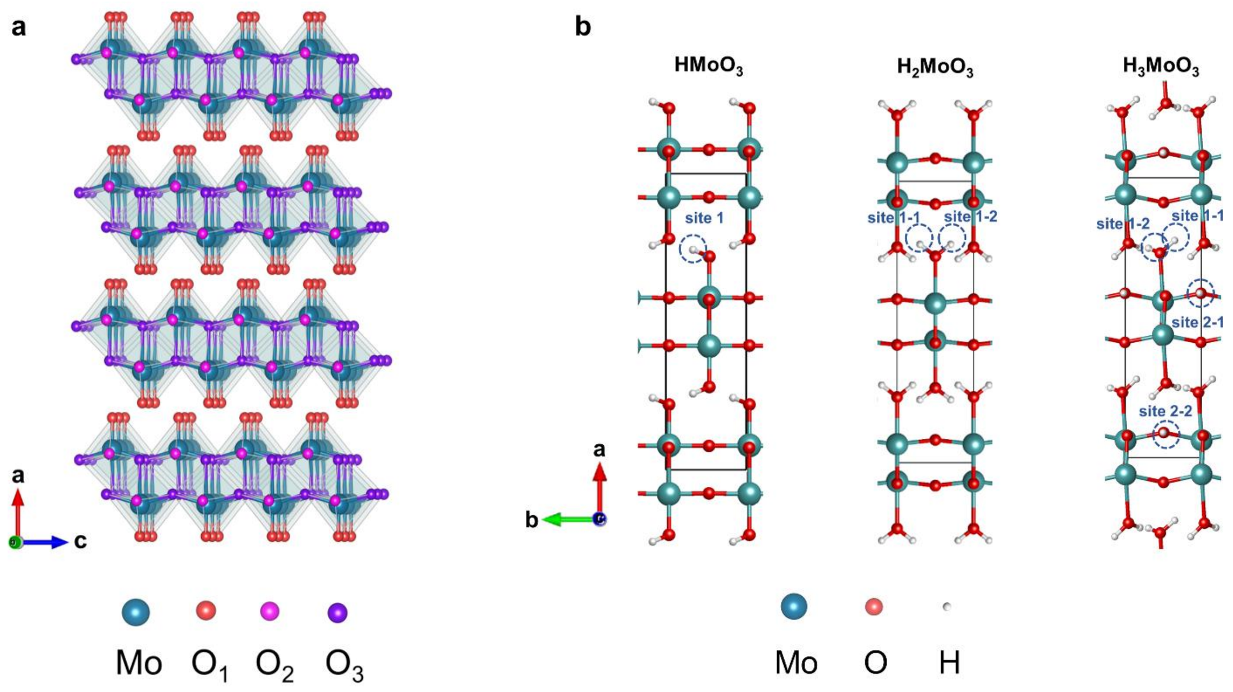

Supplementary Figure 23| The illustration of crystal structures of α-MoO_3 and H_xMoO_3 composites. a The illustration crystal structural arrangement of oxygen atoms in α-MoO_3, highlighting three distinct types: the terminating oxygen (O_1) coordinated to a single Mo atom, the bridging oxygen (O_2) coordinated to two Mo atoms, and the oxygen shared among three Mo atoms (O_3). **b** The illustration of the DFT calculation results, highlighting the probable locations of proton storage sites in H_xMoO_3.

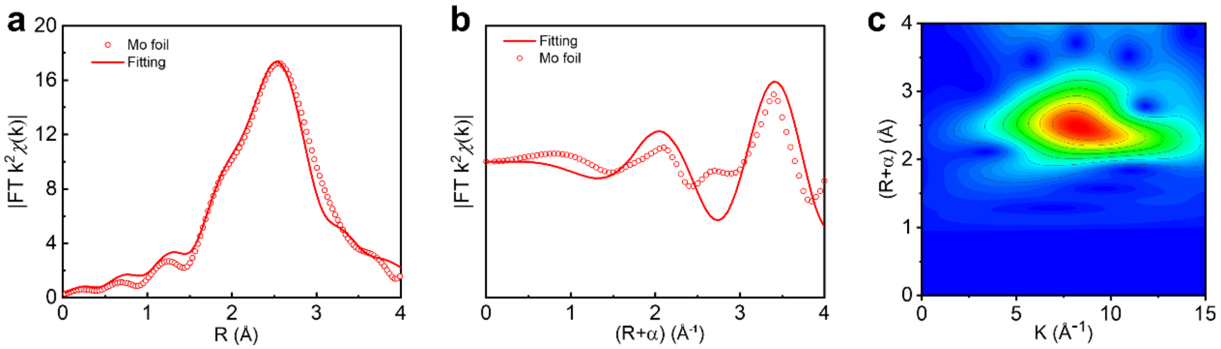

Supplementary Figure 24| The EXAFS spectra analysis of the Mo foil sample. a The FT-EXAFS spectra with the fitting result. **b** EXAFS spectra and fitting curve of Mo K-edge for Mo foil in *R* space. **c** Wavelet transform for the k^2-weighted EXAFS signal of Mo foil.

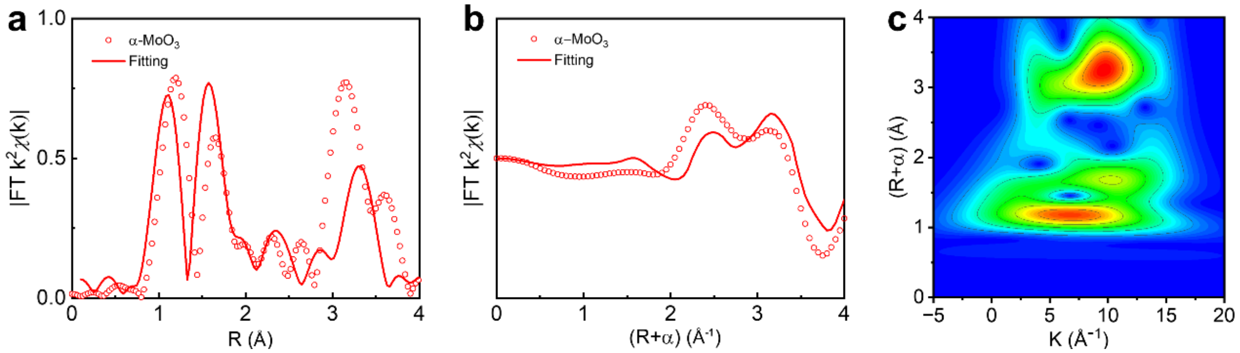

Supplementary Figure 25| The EXAFS spectra analysis of the α-MoO_3 sample. a The FT-EXAFS spectra with the fitting result. **b** EXAFS spectra and fitting curve of Mo K-edge for α-MoO_3 in *R* space. **c** Wavelet transform for the k^2-weighted EXAFS signal of α-MoO_3.

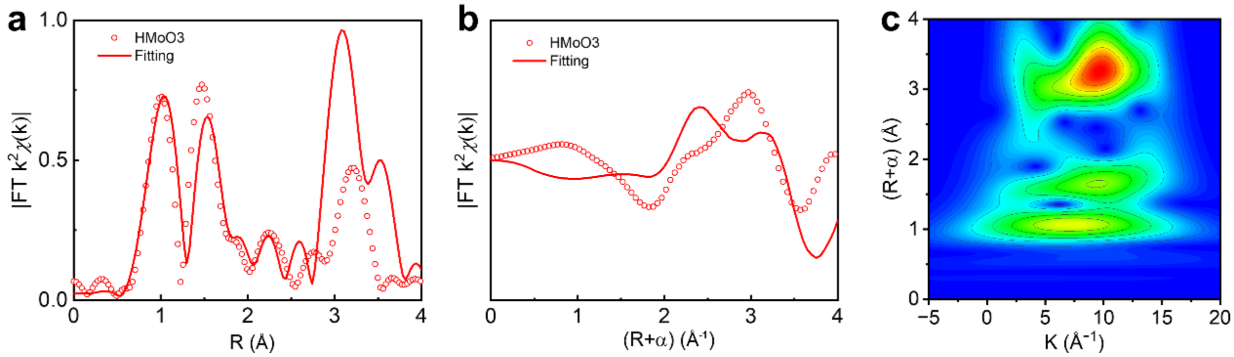

Supplementary Figure 26| The EXAFS spectra analysis of the $HMoO_3$ sample. a The FT-EXAFS spectra with the fitting result. **b** EXAFS spectra and fitting curve of Mo K-edge for $HMoO_3$ in *R* space. **c** Wavelet transform for the k^2-weighted EXAFS signal of $HMoO_3$.

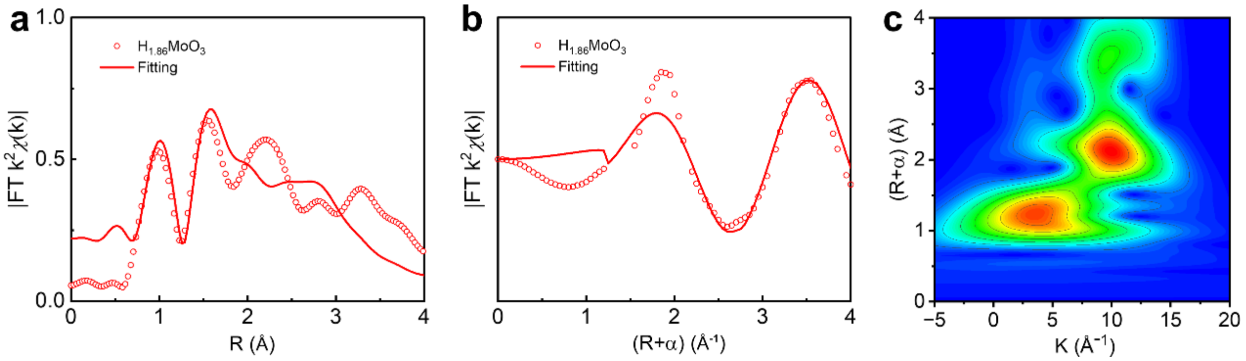

Supplementary Figure 27| The EXAFS spectra analysis of the $H_{1.86}MoO_3$ sample. a The FT-EXAFS spectra with the fitting result. **b** EXAFS spectra and fitting curve of Mo K-edge for $H_{1.86}MoO_3$ in *R* space. **c** Wavelet transforms for the k^2-weighted EXAFS signal of $H_{1.86}MoO_3$.

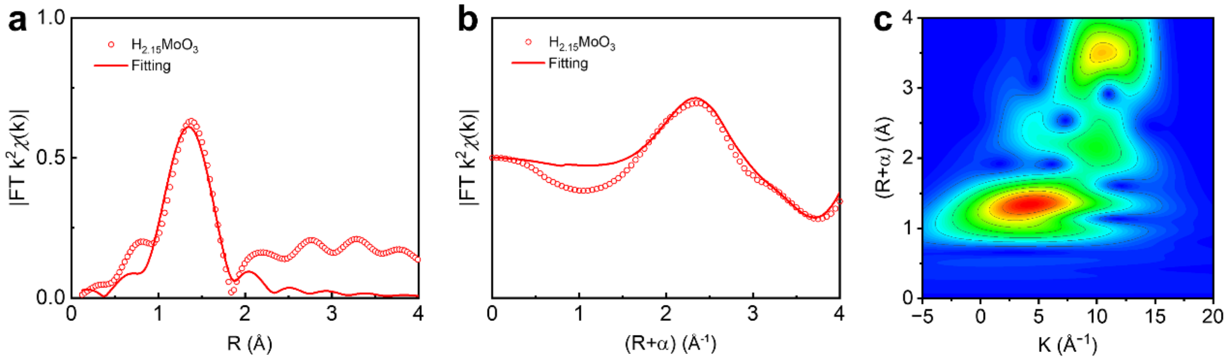

Supplementary Figure 28| The EXAFS spectra analysis of the $H_{2.15}MoO_3$ sample. a The FT-EXAFS spectra with the fitting result. **b** EXAFS spectra and fitting curve of Mo K-edge for $H_{2.15}MoO_3$ in *R* space. **c** Wavelet transforms for the k^2-weighted EXAFS signal of $H_{2.15}MoO_3$.

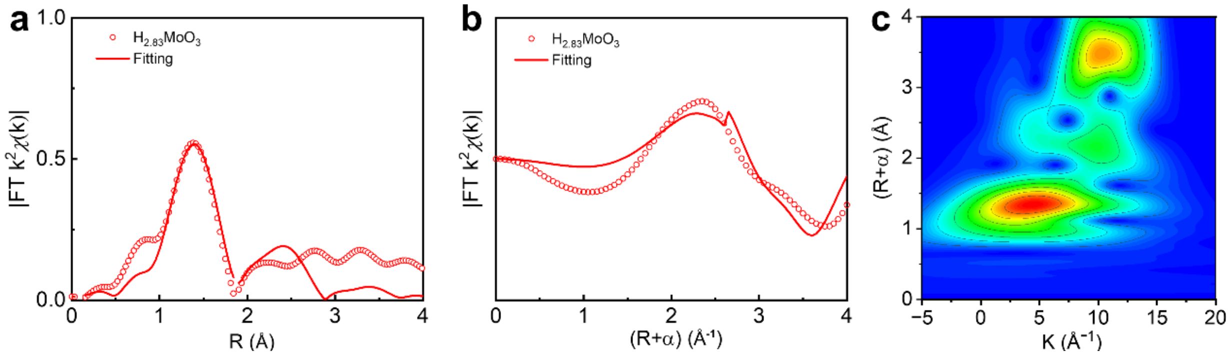

Supplementary Figure 29| The EXAFS spectra analysis of the $H_{2.83}MoO_3$ sample. a The FT-EXAFS spectra with the fitting result. **b** EXAFS spectra and fitting curve of Mo K-edge for $H_{2.83}MoO_3$ in *R* space. **c** Wavelet transforms for the k^2-weighted EXAFS signal of $H_{2.83}MoO_3$.

Supplementary Table 2 Structural parameters extracted from the EXAFS fitting.

Sample	Shell	*CN*	*R* (Å)	σ^2 (10^{-3}Å^2)	ΔE_0 (eV)	*R* factor
Mo foil	Mo-Mo	12 (fixed)	2.85±0.01	1.7±1.6	6.1±1.4	0.009
α-MoO3	Mo-O	5.6±0.7	2.18±0.01	1.1±1.2	4.9±3.2	0.014
$HMoO_3$	Mo-O	4.9±1.3	2.16±0.01	11.1±7.9	4.9±1.9	0.009
$H_{1.86}MoO_3$	Mo-O	4.4±0.2	2.10±0.00	1.1±1.2	2.7±8.1	0.020
$H_{2.15}MoO_3$	Mo-O	3.9±0.5	2.02±0.01	12.7±2.9	1.3±1.9	0.014
$H_{2.83}MoO_3$	Mo-O	3.2±0.3	2.01±0.01	2.3±8.1	1.5±2.5	0.013

CN represents the coordination number; *R* represents the interatomic distance; σ^2 represents the Debye-Waller factor; ΔE_0 represents the edge-energy shift. *R* factor: goodness of fit.

Supplementary Table 3 The theoretical Mo-O distances of α-MoO_3.

Mo-O	Distances (Å)
Mo-O_1	1.678
Mo-O_2^{I}	1.738
Mo-O_2^{II}	2.244
Mo-O_3^{I}	1.949
Mo-O_3^{II}	1.949
Mo-O_3^{III}	2.327

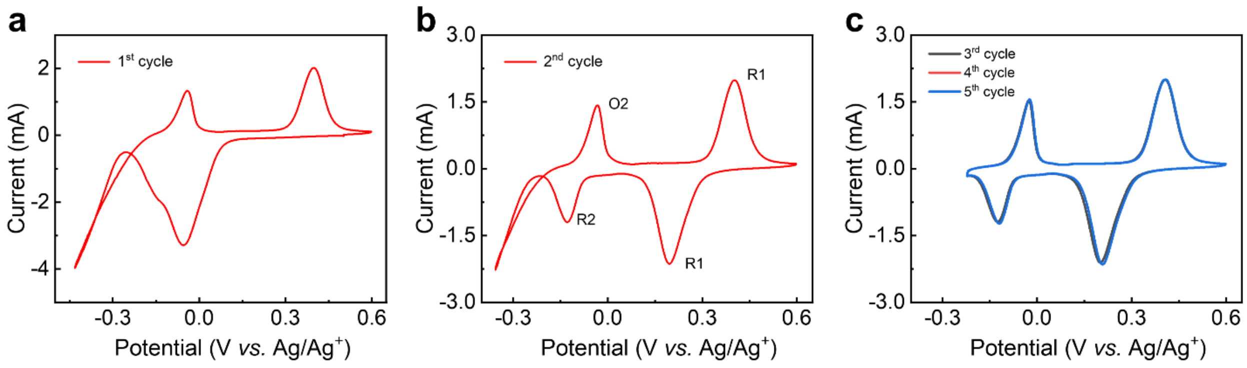

Supplementary Figure 30| CV curves of the α-MoO_3 electrode using the non-aqueous acid electrolyte at the scan rate of 1 mV s^{-1}: 1M trifluoromethanesulfonic acid in an ionic liquid 1-Ethyl-3-MethylImidazolium Bis (TriFluoroMethylSulfonyl) Imide **a** The first cycle, **b** The second cycle, and **c** the third to fifth cycles. The experiment was conducted in an Argon-filled glovebox.

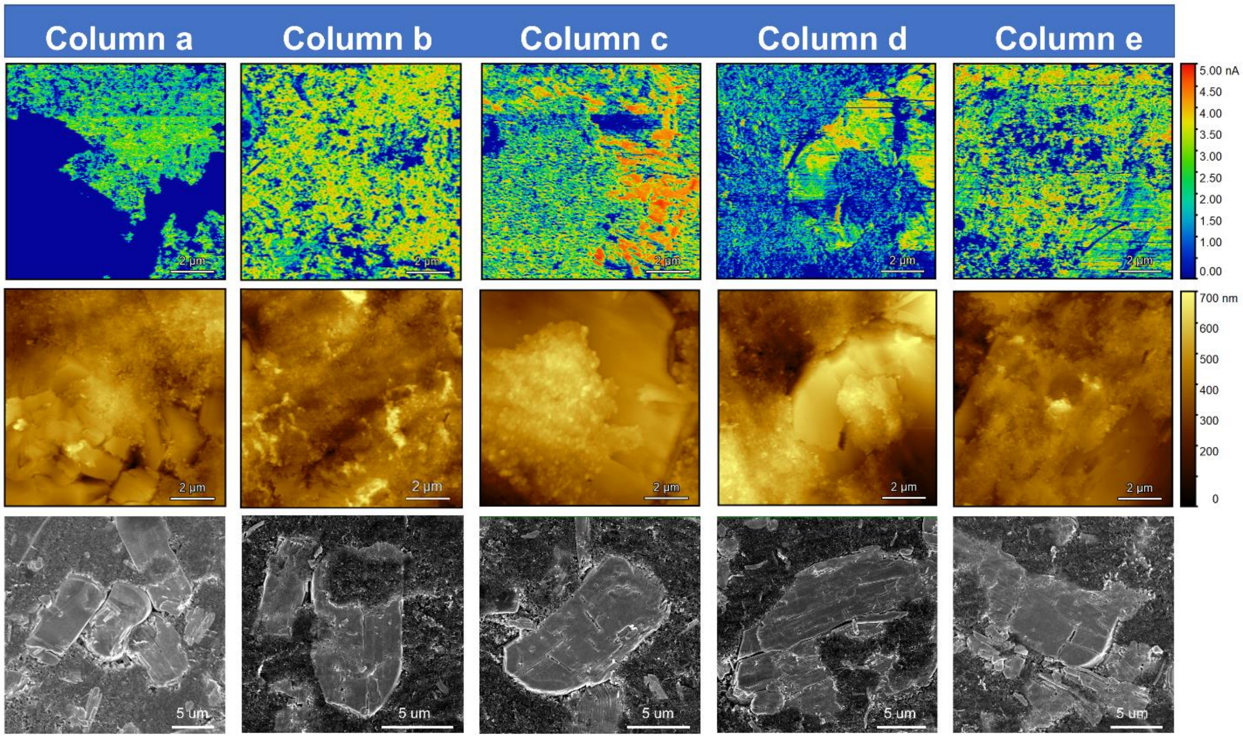

Supplementary Figure 31| Current-sensitive atomic force microscopy (CS-AFM) analysis of different MoO_3 electrodes. a pristine electrode, **b** discharged to 0.35 V, **c** discharged to -0.15 V, **d** discharged to -0.45 V, **e** discharged to -0.65 V. Each column from top to down: the current response of the MoO_3 electrode, the topographical AFM images of the MoO_3 electrode, and the SEM image of the MoO_3 electrode.

Supplementary Note: The α-MoO_3 electrode was prepared by casting the slurry of micro-sized α-MoO_3, Super C65 conductive carbon, and polyvinylidene fluoride (ratio: 8:1:1) on the titanium foil. After drying in the vacuum oven at 60 degrees for 24 hours, the electrodes were pressed under 5 MPa using a hydraulic press machine to reduce the surface roughness. Then the α-MoO_3 electrodes were discharged to different states using a three-electrode Swagelok system. After washing and drying, the current-sensitive atomic force microscopy (CS-AFM) analysis was performed in Tunneling Current AFM mode on the Dimension Icon Atomic Force Microscope system. The current mapping was performed with a PtIr-coated silicon probe (SCM-PIT-V2, Bruker) and a topographical scanner (scanning area: 10 x 10 μm^2) at a 0.5 Hz scan rate. -0.5 V DC bias was applied on the α-MoO_3 electrodes during measurements.

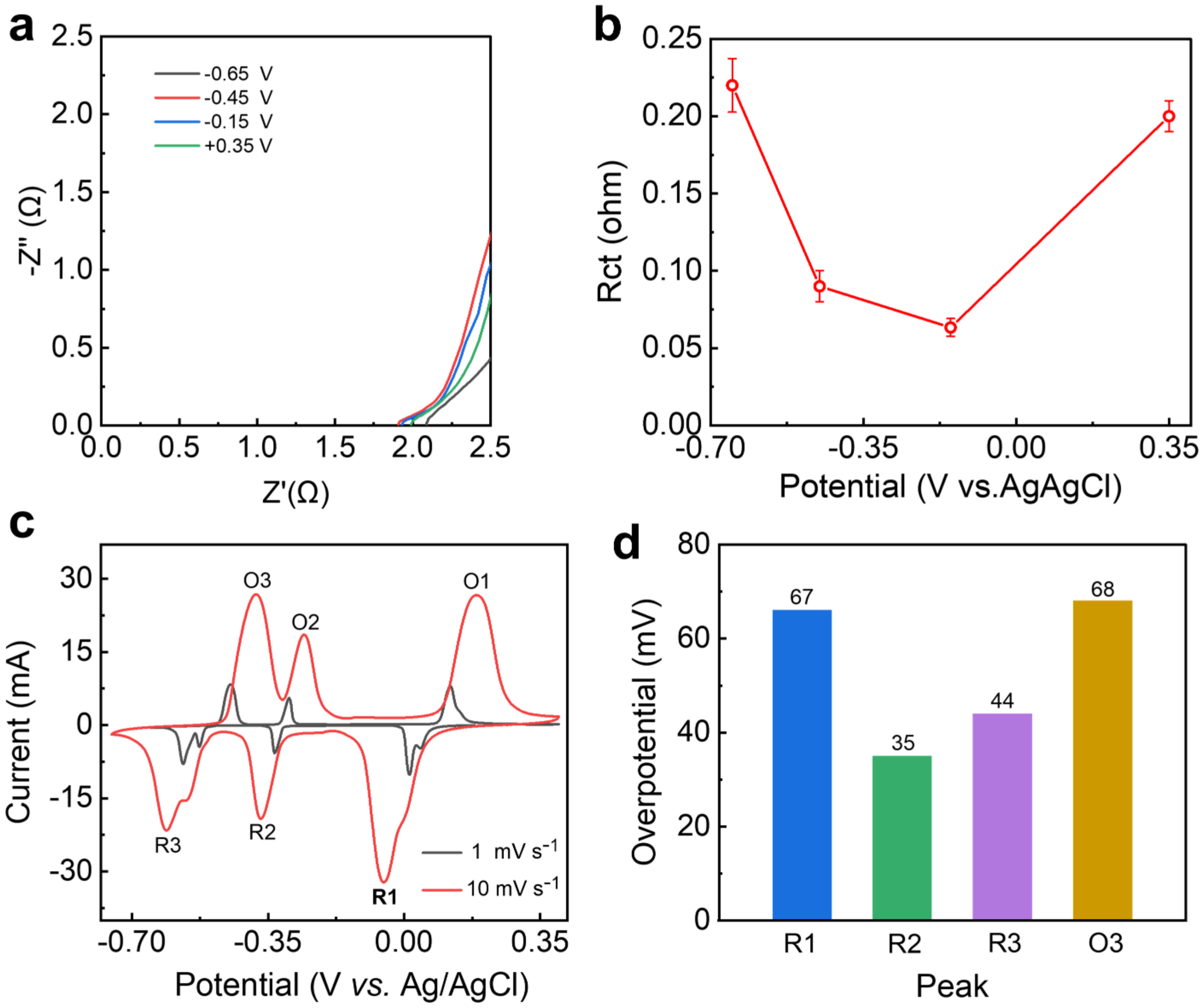

Supplementary Figure 32| Electrochemical performance of MoO_3 electrode using the PSL electrolyte. a The electrochemical impedance spectroscopy of α-MoO_3 electrodes at different discharge states. **b** the charge transfer resistance (R_{ct}) of α-MoO_3 electrodes at different discharge states (the error bars were derived from three times of tests using different samples). **c** The CV curves of the α-MoO_3 electrode recorded using PSL electrolyte at different scan rates. **d** The overpotential of different redox peaks (from 1 mV s^{-1} to 10 mV s^{-1}) derived from the CV curves in **c**.

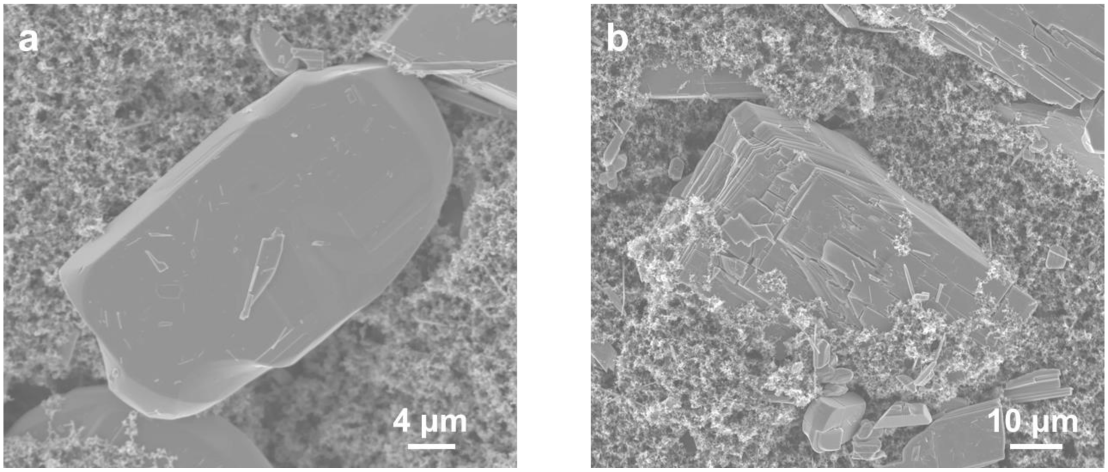

Supplementary Figure 33| The SEM images of **a** pristine α-MoO_3 electrode, and **b** MoO_3 electrode after the first GCD cycle using PSL electrolyte.

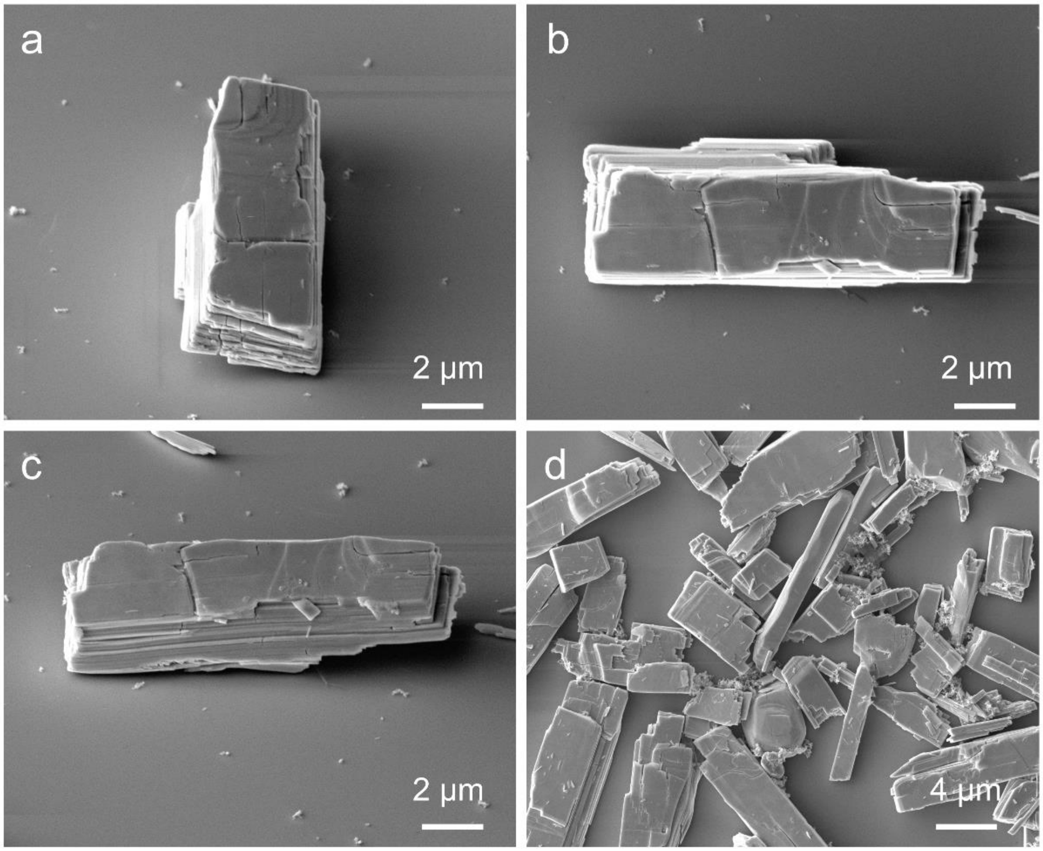

Supplementary Figure 34| SEM morphology characterization of MoO_3 particles. a to **c** The SEM images of MoO_3 particles after the initial redox peak in the first discharge process using PSL electrolyte, which is used for Focused Ion Beam (FIB) cutting. **d** The SEM image of MoO_3 particles at low magnification.

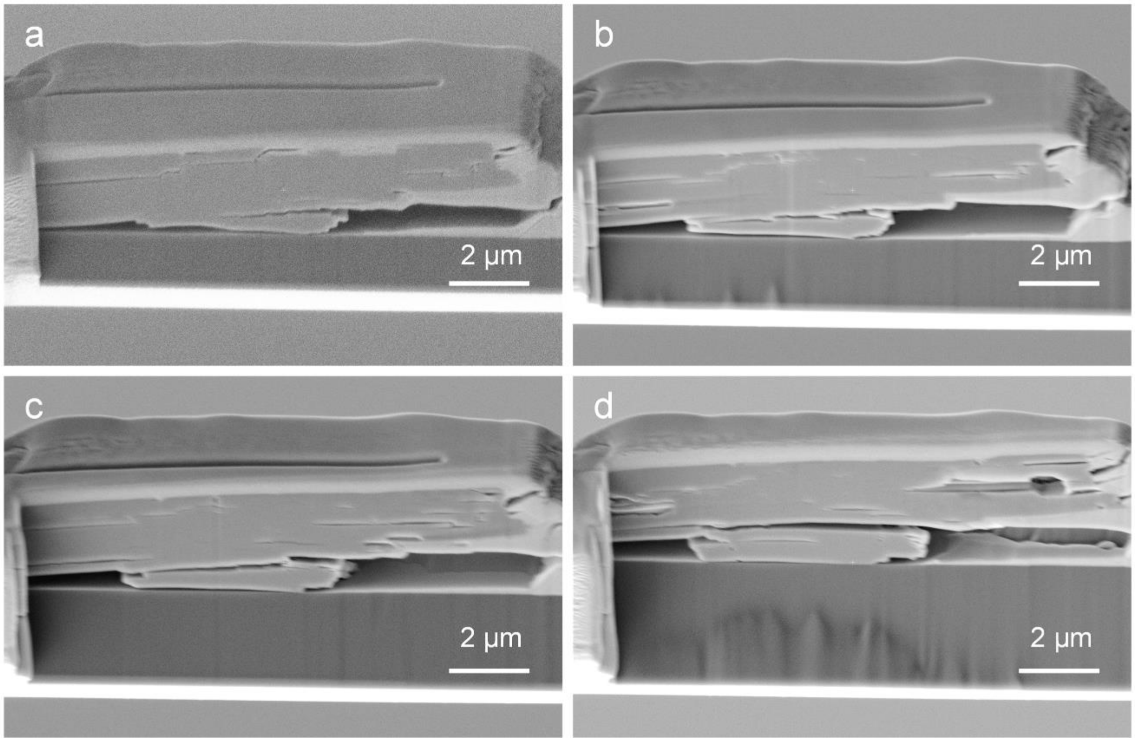

Supplementary Figure 35| The SEM images of MoO_3 particle after the initial redox peak in the first discharge process using PSL electrolyte. a-d Sectional view SEM image of MoO_3 electrode after the FIB cutting on the left side (1 μm cutting each from **a** to **d**).

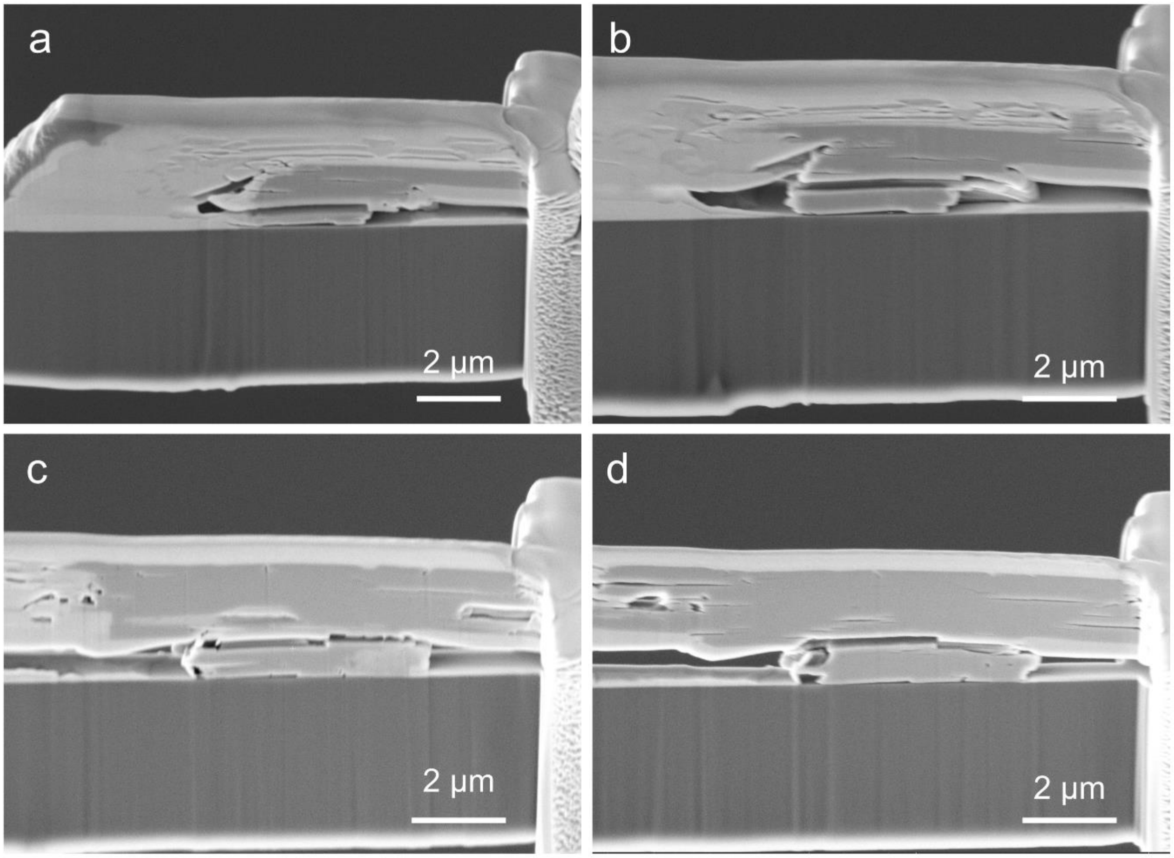

Supplementary Figure 36| The SEM images of MoO_3 particle after the initial redox peak in the first discharge process using PSL electrolyte. a-d Sectional view SEM image of MoO_3 electrode after the FIB cutting on the right side (1 μm cutting each from **a** to **d**).

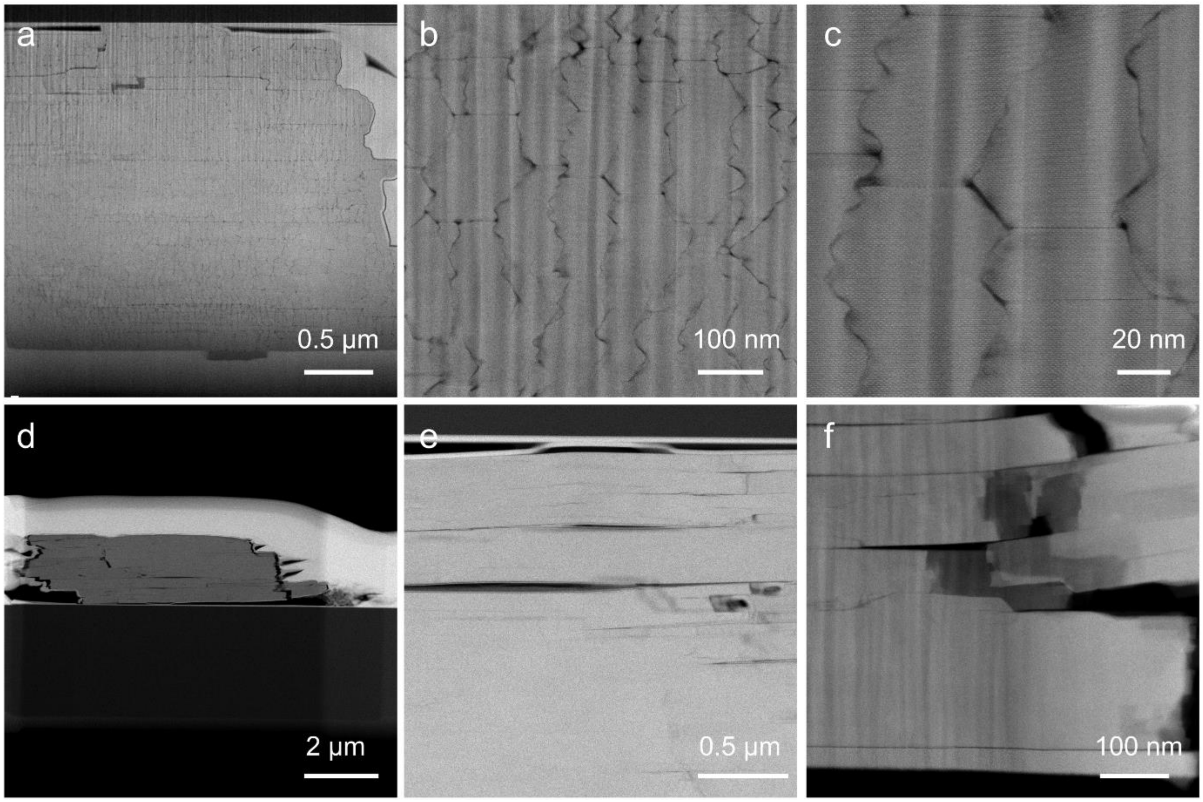

Supplementary Figure 37| TEM morphology characterization of $HMoO_3$ particles. a to **c** HR-STEM images of the $HMoO_3$ lamella sample with different magnifications viewed along the [001] direction. **d** to **f** STEM images of the $HMoO_3$ lamella sample with different magnifications viewed along the [010] direction.

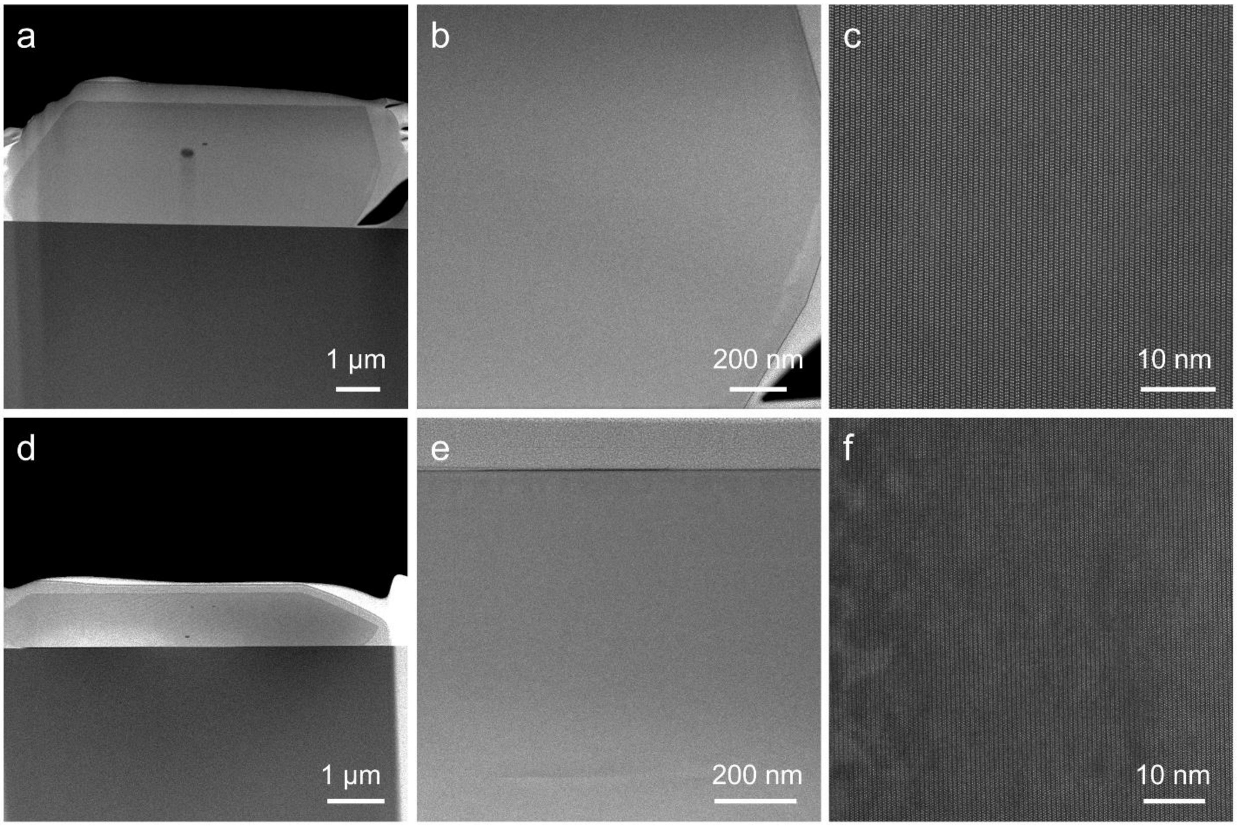

Supplementary Figure 38| TEM morphology characterization of α-MoO_3 particles. a to **c** HR-STEM images of the α-MoO_3 lamella sample with different magnifications viewed along the [001] direction. **d** to **f** STEM images of the α-MoO_3 lamella sample with different magnifications viewed along the [010] direction.

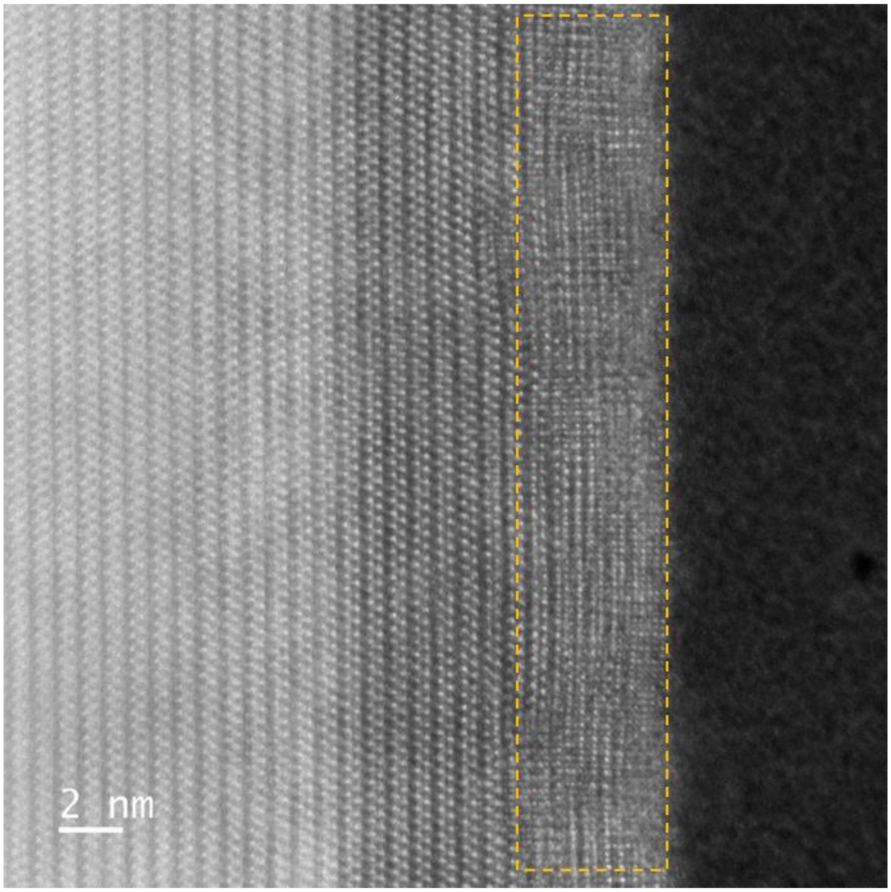

Supplementary Figure 39| High-resolution HADDF image of α-MoO_3 particle surface layers, with the inset (orange box) displaying the atomically dense surface oxide layers of α-MoO_3 particles.

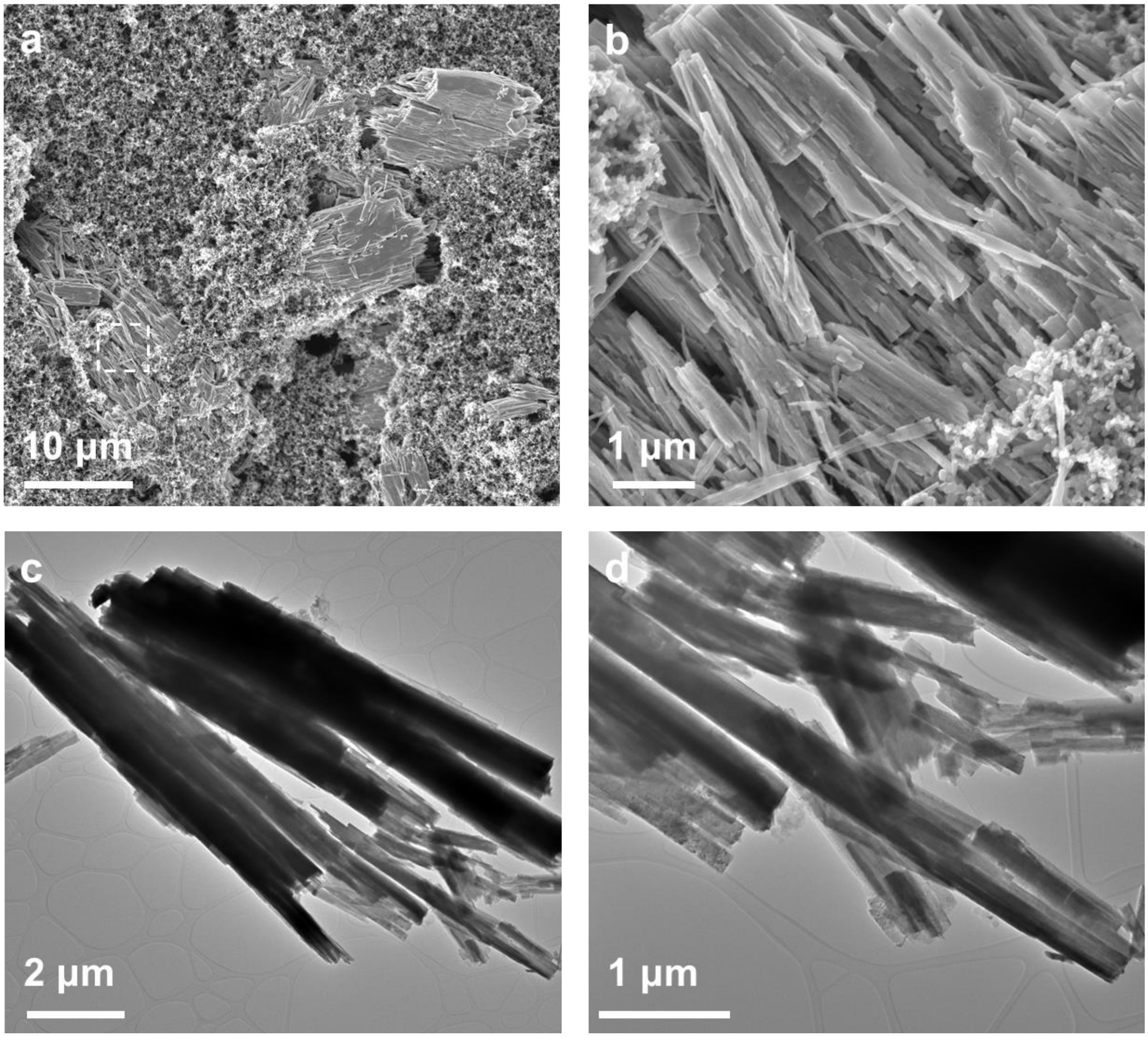

Supplementary Figure 40| SEM and TEM morphology characterization of MoO_3 particles. **a** SEM image of MoO_3 electrode after the 20,000 GCD cycles using PSL electrolyte. **b** the zoom-in HR-SEM image of the white box area in **a**. **c** and **d** TEM images of the MoO_3 particle after 20,000 GCD cycles.

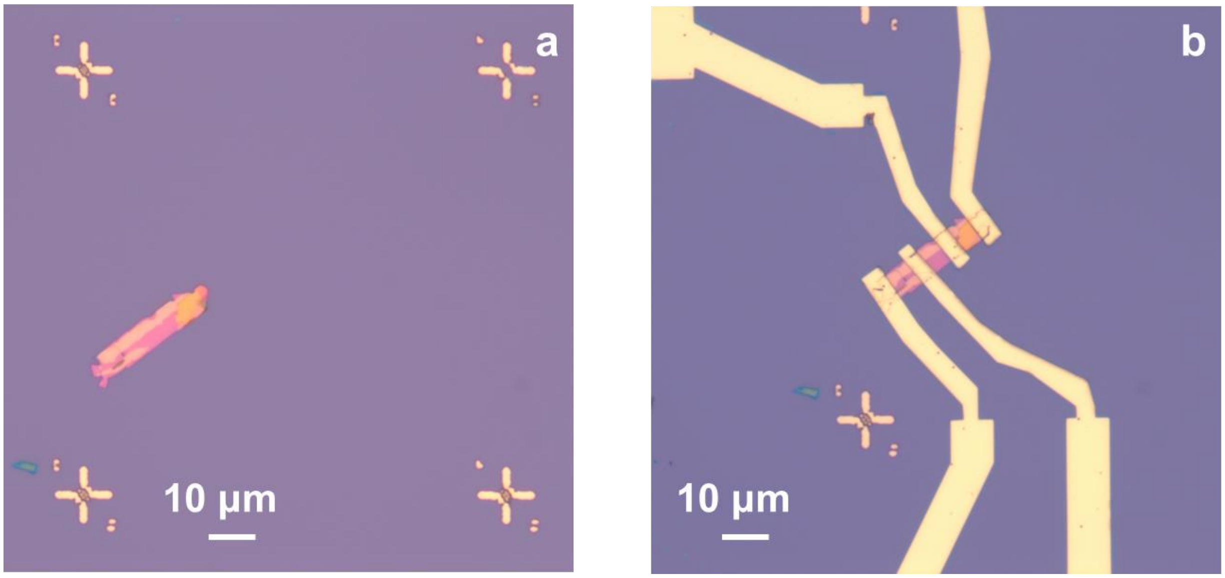

Supplementary Figure 41| The photo of 20,000 GCD cycled MoO_3 particles before **a** and after **b** modified with conducting wires.

Supplementary Note: Resistance measurements were carried out with a four-terminal configuration. The sample was put onto a SiO_2 (300 nm)/Si substrate, and the thickness was verified using an atomic force microscope (Dimension Icon). Subsequently, the electrodes were patterned via electron-beam lithography (Crestec-9000), followed by e-beam evaporation of Ti (10 nm)/Au (90 nm) metals. Room-temperature electrical measurements were performed on Keithley 4200 semiconductor parameter analyzer. Low-temperature electrical measurements were performed in a Physical Property Measurement System (Quantum Design). The fitting curves of resistance-temperature data for the conductor and the semiconductor followed the below equations:

Equation 1: $R(T) = R(\mathrm{T}_0)(1 + \alpha \Delta T)$ for conductor.

Equation 2: $R_i = \frac{L}{AC} e^{\left[\frac{Eg}{2K_B T}\right]}$ for semiconductor.[1]

Where L is the length, A is the cross-sectional area, C is a constant, E_g is the band gap.

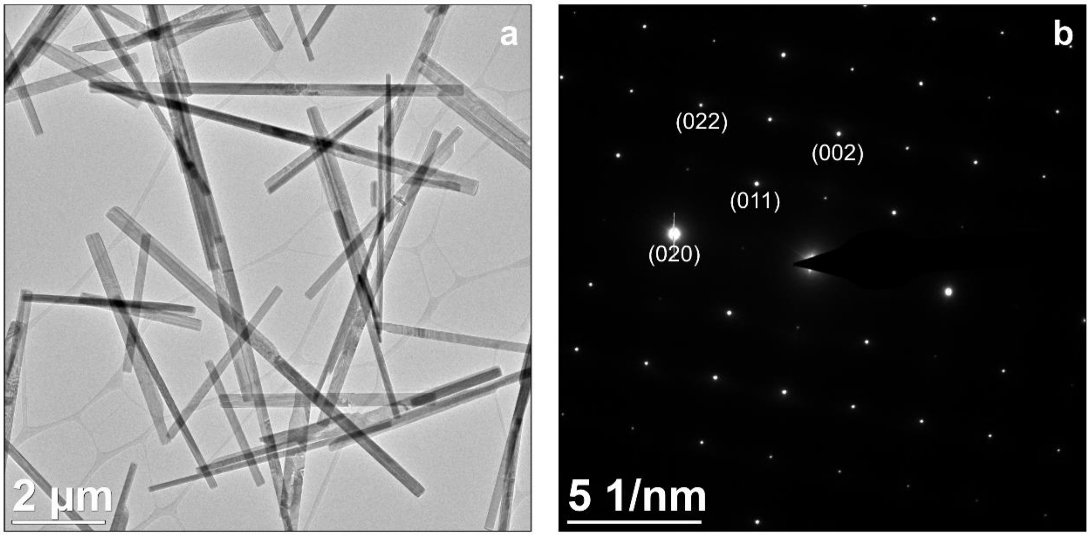

Supplementary Figure 42| Structure characterizations of the α-MoO_3 nanobelts. a TEM image of hydrothermal synthesized α-MoO_3 nanoribbon. **b** SAED pattern of MoO_3 nanoribbon with a zone axis along [100].

Supplementary Note: The α-MoO_3 nanoribbon was prepared by the reported hydrothermal method.[2] Typically, 1.0 g of $(NH_4)_6Mo_7O_{24}\cdot 4H_2O$ was dissolved in 25 mL de-ionized (DI)water, and 10 M HNO_3 was added in to adjust the pH to 2. After stirring for 30 minutes, the solution was transferred to the 50 mL autoclave and was kept at 180 °C for 16h. The obtained powder was washed with DI water several times and dried at 60 °C overnight.

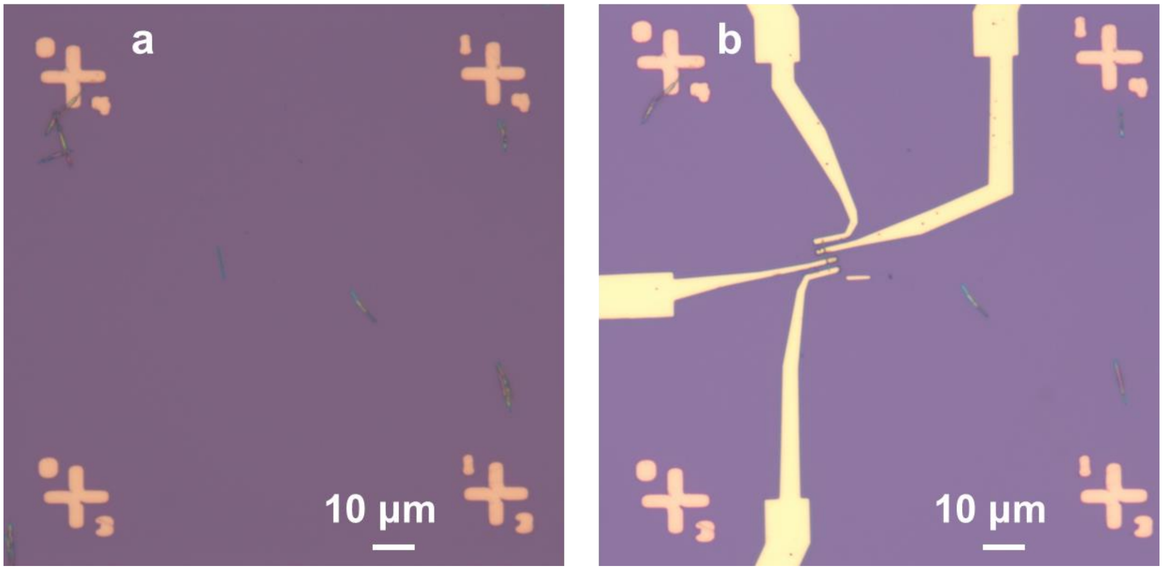

Supplementary Figure 43| The photos of hydrothermal synthesized α-MoO_3 nanoribbon before **a** and after **b** modified with conducting wires.

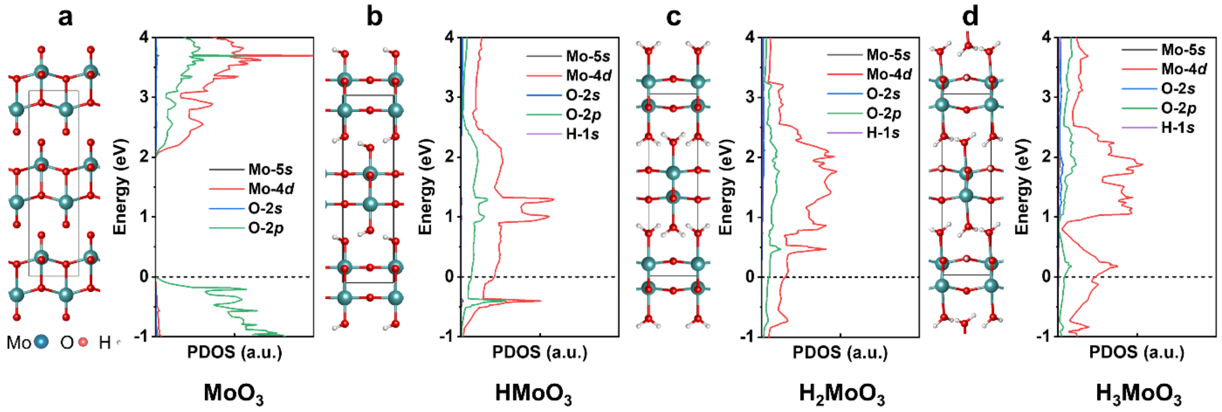

Supplementary Figure 44| Evolution of PDOS with increasing H^+ intercalation levels, suggesting the transformation of semiconducting α-MoO_3 to metallic H_xMoO_3. **a** pristine α-MoO_3. **b** $HMoO_3$. **c** H_2MoO_3. **d** H_3MoO_3.

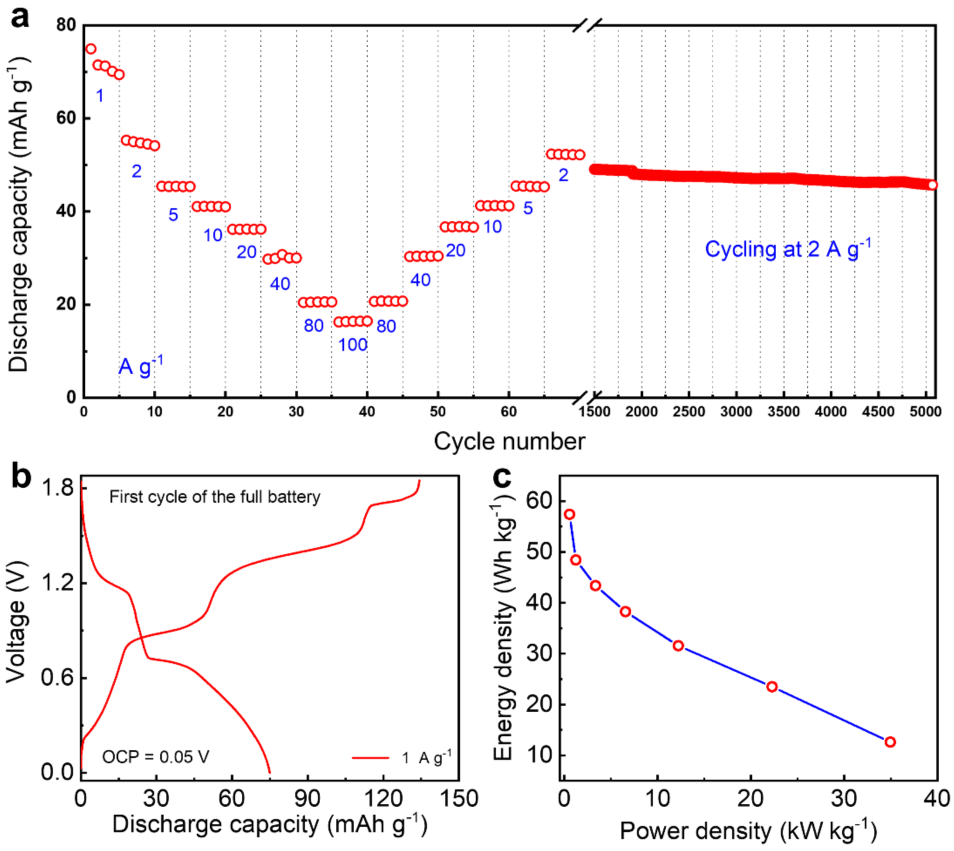

Supplementary Figure 45| Electrochemical performance of α-MoO_3/CuFe-PBA full battery. **a** Rate performance of full battery at the current density of 1 to 100 A g^{-1}. **b** The first GCD curve of the full battery, the as-assembled full cell displayed the open circuit potential (OCP) of 0.05 V. **c** Energy density and power density of the full battery. The current density and specific discharge capacity were calculated based on the mass of both cathode and anode active materials.

References

1 Alenitsyn, A. G., Butikov, E. I., & Kondratyev, A. S. *Concise Handbook of Mathematics and Physics.* (CRC Press, Boca Raton, 1997).

2 Lou, X. W. & Zeng, H. C. Complex alpha-MoO_3 nanostructures with external bonding capacity for self-assembly. *J. Am. Chem. Soc.* **125**, 2697-2704 (2003).
